# Cytotoxic and pro-apoptotic effects of botanical drugs derived from the indigenous cultivated medicinal plant *Paris polyphylla* var. *yunnanensis*

**DOI:** 10.3389/fphar.2023.1100825

**Published:** 2023-01-26

**Authors:** Xiu-Xiang Yan, Yan-Qiang Zhao, Yun He, Terd Disayathanoowat, Hataichanok Pandith, Angkhana Inta, Li-Xin Yang

**Affiliations:** ^1^ Key Laboratory of Economic Plants and Biotechnology, Kunming Institute of Botany, Chinese Academy of Sciences, Kunming, Yunnan, China; ^2^ Department of Biology, Faculty of Science, Chiang Mai University, Chiang Mai, Thailand; ^3^ College of Forestry and Vocational Technology in Yunnan, Kunming, Yunnan, China; ^4^ Lijiang Yunxin Green Biological Development Co., Ltd., Lijiang, Yunnan, China

**Keywords:** *Paris polyphylla* var. *yunnanensis*, indigenous medicine knowledge, saponins, cytotoxic effects, pro-apoptotic effects

## Abstract

**Background:** Cancer is one of the top two leading causes of death worldwide. Ethnobotanical research, it is one of methods, which is able to discover effective anticancer drugs based on “prototype” of indigenous people’s historical experiences and practices. The rhizomes of *Paris polyphylla* var. *yunnanensis* (Franch.) Hand.-Mazz. have been used as botanical drugs to treat cancer by Yi, Bai, Dai, and Naxi ethnic groups in Yunnan, China, where this species is widely cultivated in a large scale in Yunnan.

**Materials and methods:** To identify the substances of anticancer activities based on indigenous medicine knowledge, chromatography was performed to separate saponins from the rhizomes of *P. polyphylla* var. *yunnanensis*, followed by spectroscopy to determine the structure of six isolated saponins. The cytotoxicity of five extracts and six pure compounds were evaluated by MTS method. Quantitative determination of total saponins of *P. polyphylla* var. *yunnanensis* was analyzed by HPLC. Cell cycle assay, apoptosis assay, and mitochondrial membrane potential were used to evaluate the pro-apoptotic activity *in vitro*.

**Results:** Five extracts and six pure saponins showed significant inhibitory cytotoxic activities of three human liver cancer cell lines (SMMC-7721, HepG2, and SK-HEP-1) and one non-small-cell lung cancer cell line (A549). The contents of Paris saponins I, II, and VII were 6.96% in the rhizomes of *P. polyphylla* var. *yunnanensis*, much higher than Chinese Pharmacopoeia standards (0.6%). Six saponins induced significant apoptosis and cell cycle arrest in three human cancer cell lines (A549, SMMC-7721, and HepG2), which was associated with the loss of mitochondrial membrane potential.

**Conclusion:** The result of this study support that cultivated *P. polyphylla* var. *yunnanensis* could be a substitute for wild resource as an anticancer medicine based on indigenous medicine knowledge.

## 1 Introduction

Lung cancer remains the leading cause of total cancer death, with an estimated 18% mortality, followed by colorectal (9.4%) and liver (8.3%) cancers according to global cancer statistics for 2020 (Sung et al., 2021). Hepatocellular carcinoma (HCC) is a major type of primary liver cancer and is the third leading cause of cancer-related deaths worldwide (Yang and Roberts, 2010; Liu et al., 2013), while lung cancer has the second highest morbidity rate (11.4%) next only to female breast cancer (11.7%). Lung cancer can be divided into small-cell lung cancer and non-small-cell lung cancer (NSCLC) based on cell type, the latter being responsible for over 80% of all lung cancers (Ferlay et al., 2015; Sui et al., 2016). Although cancer treatment and prognosis vary with the type of cancer, the most widely available treatments for the above two cancer types are surgery, chemotherapy, and radiotherapy. However, these treatments and their clinical effects do not always lead to a positive outcome (Yang et al., 2012). In recent years, cancer research has embraced complementary approaches such as targeted therapy inspired by indigenous medicine knowledge (Liu et al., 2021; Bi et al., 2022; Liu et al., 2022) as well as anticancer treatments by immunotherapy (Liu et al., 2018). Novel anticancer drugs with high effects, low toxicity, and low cost are urgently needed to treat these two cancers. One potential source of untapped knowledge is folk remedies, such as those described in TCM, although the effects of these traditional ethnic treatments and the identity of the underlying bioactive substance remain to be established and fully explored.

The genus *Paris* of the family Melanthiaceae encompasses 26 species based on current taxonomy, also known as “Chong-Lou” in Chinese (Li, 1998; Wang et al., 2015; Wang et al., 2017; Wang et al., 2018; Pei et al., 2020; Ji, 2021) and “余猫瀑 (yuq ma pvl)” in the Naxi language. The dried rhizome of *Paris polyphylla* var. *yunnanensis* (Franch.) Hand.-Mazz. and *Paris polyphylla* var. *chinensis* (Franch.) Hara are widely used in TCM and are recorded in the Chinese Pharmacopoeia (Wang et al., 2015; National Pharmacopoeia Committee, 2020; Yan et al., 2021). In fact, the dried rhizomes of these plants (Rhizoma Paridis) are an indispensable ingredient of seven preparations recorded in the Chinese Pharmacopoeia (2020). One of these seven preparations is “Qizhen capsule” (芪珍胶囊), which has traditionally served as an adjuvant therapy for lung, breast, and gastric cancer (Huang et al., 2011; Qin et al., 2018; National Pharmacopoeia Committee, 2020). *P. polyphylla* var. *yunnanensis* is widely used as a botanical drugs by eight ethnic minorities in China, constituting one ingredient from 62 recipes effective for the main treatment of multiple human diseases and afflictions, such as tumors, skin injury, poisoning, virus infections, diseases, and ailments affecting connective tissues and the respiratory, digestive, urogenital, and musculoskeletal systems. More specifically, four ethnic groups—the Yi, Bai, Dai and Naxi people—have used *P. polyphylla* var. *yunnanensis* to treat cancer (Yan et al., 2021). While TCM has been tested in human trials, the underlying pharmacological mechanism has not been clearly established, thereby limiting its potential benefits for human health.

A survey of the chemical structures of the few traditional compounds that have been isolated and identified has revealed that the main active components are often steroidal saponins (Huang and Zhou, 1962; Chen and Zhou, 1987; Kang et al., 2012; Wu et al., 2012; Qin et al., 2013; Wu et al., 2013; Wei et al., 2014; Qin et al., 2016; Ding et al., 2021), which are able to fight off cancer, furuncles, carbuncles, abscesses, bacterial infections, and inflammation, as well as stop bleeding (Yan et al., 2021). Indeed, metabolite studies in the *Paris* genus have led to the isolation and identification of 323 compounds, consisting of steroidal saponins (168), triterpenes (33), flavonoids (30), cholestanols (8), C-21 steroids (11), phytosterols (12), phytoecdysones (11), and other secondary metabolites (50). A total of 177 compounds have been isolated in *P. polyphylla* var. *yunnanensis*, of which 112 were steroidal saponins (Wei et al., 2014; Zhou et al., 2022). Plant extracts from the *Paris* genus exhibit anti-tumor, hemostasis, analgesic, anti-inflammatory, and antibacterial effects (Deng et al., 2008; Qin et al., 2012; Qin et al., 2016; Zhang et al., 2016; Ding et al., 2021; Yan et al., 2021). For instance, the anticancer activity of total steroidal saponins from the rhizomes and aboveground parts of *P. polyphylla* var. *yunnanensis* is demonstrated against five human cancer cell lines (SW-480, SMMC-7721, HL-60, MCF-7, and A-549) (Qin et al., 2018). However, the identity of the active compounds in the planting resource of this species has not been established according to promising ethnobotanical results. Moreover, the diversity in saponin chemical structures (Ding et al., 2021; Yan et al., 2021) makes their identification challenging. Here, extracted, isolated, purified, and identified individual compounds and established their anti-proliferation potential against several human cancer cell lines, suggesting new promising metabolites to fight cancer.

This study is mainly intended to validate the ethnopharmacological use of *P. polyphylla* var. *yunnanensis*, by four ethnic groups in China. An interdisciplinary approach was used to identify and quantify the active steroidal saponins from ethanol extract. The ethanol extracts were further separated to obtain pure compounds, which were identified by nuclear magnetic resonance and electrospray ionization–mass spectrometry. Anticancer properties of five crude extracts and six pure saponins were evaluated by *in vitro* cell viability assays against three human liver cancer cell lines and one non-small-cell lung cancer cell line. Furthermore, the effects of six pure saponins on apoptosis and cell cycle arrest on cancer cell lines were investigated, some of which were sensitive to the saponins.

## 2 Materials and methods

### 2.1 Traditional anticancer records

Four ethnic groups—the Yi, Bai, Dai, and Naxi people—have used *P. polyphylla* var. *yunnanensis* to treat cancer (Yan et al., 2021). The Yi ethnic group mix about 10 g (fresh weight) the rhizomes of *P. polyphylla* var. *yunnanensis* and *Rohdea chinensis* (Baker) N. Tanaka and make it into a decoction or use 3 g (dry weight) of the same mixture for the treatment of gynecological tumors. The Bai ethnic group employs 15 g each of a powder from *P. polyphylla* var. *yunnanensis*, *Viola philippica* Cav., *Hedyotis diffusa* Willd., and *Scutellaria barbata* D. Don and make it into a decoction for use once a day for 1–2 weeks to treat stomach and esophageal cancers. The Naxi ethnic group uses powder from *P. polyphylla* var. *yunnanensis* (20 g), *H. diffusa* Willd. (50 g), *S. barbata* D. Don (50 g), and *Engleromyces goetzi* P. Henn. (20 g) to prepare a decoction, which is consumed every day over 1–2 months for cancer treatment; the types of cancer were not specified. For the Dai ethnic group, there was a record treatment for cancer, but the prescription and type of cancer are unknown (Yan et al., 2021).

There are five types of cancer treatments using *P. polyphylla* var. *yunnanensis* including brain neoplasms and thyroid, laryngeal, gastric, and lung cancer, which have been recorded in the book of Yunnan Kang Ai Zhong Cao Yao (Hu and Xuan, 1982). There are 10 kinds of traditional Chinese medicine recipes for lung cancer, each using 30 g of powder as the basis for a decoction, comprising *P. polyphylla* var. *yunnanensis*, *C. lacryma-jobi*, *S. scandens*, *H. cordata*, *Wolfiporia cocos* (F. A. Wolf) Ryvarden & Gilb., *Polyporus umbellatus*, *M. tenacissima*, *Asparagus densiflorus* (Kunth) Jessop, *Platycodon grandiflorus* (Jacq.) A. DC., and *Sageretia thea* (Osbeck) Johnst. in various amounts. These anticancer prescriptions that include *P. polyphylla* var. *yunnanensis*, but the pharmacological and chemical properties of this species that could be effective for treating anticancer are unclear.

### 2.2 General experimental procedure

Deionized water was used throughout the study. Ethanol was analytical grade. ^1^H and ^13^C NMR spectra were collected on an Avance III 600 spectrometer (Bruker, Switzerland) with tetramethylsilane (TMS) as an internal standard in pyridine-*d*_5_ (Cambridge Isotope Laboratories, Inc.). An ultra-high-performance liquid chromatograph connected to a triple quadrupole tandem mass spectrometer (UPLC-Xevo TQ MS, Waters, United States) was used to obtain electrospray ionization–mass spectrometry (ESI-MS) data. Silica gel (200–300 mesh, Qingdao Haiyang Chemical Co., Ltd., Qingdao, China), a macroporous resin column (D101, Jiangsu Donghong Chemical Co., Ltd., China), and thin-layer chromatography (TLC) plates (Qingdao Haiyang Chemical Co., Ltd., China) were used to monitor the compound. The TLC spots were visualized under ultraviolet (UV) light at 254 nm and stained by spraying the TLC plates with 5% (v/v) H_2_SO_4_ in alcohol followed by heating. Semi-preparative high-performance liquid chromatography (HPLC) analysis was performed on an Agilent 1260 series system (Agilent Technologies, United States) equipped with a diode array detector, and preparative HPLC was conducted with a Hanbon series system (Jiangsu Hanbon Science & Technology Co., Ltd., China) equipped with a UV detector, using two types of C18 columns produced by Agilent (Zorbax SB-C18 column, 5 μm; ϕ 9.4 × 250 mm and ϕ 4.6 × 250 mm).

### 2.3 Plant materials

As an identification of Good Agriculture Practice (GAP) and geographical indication products, the rhizomes of *P. polyphylla* var. *yunnanensis* which is native and local distribution (called “Daodi” medicine in Chinese) were obtained from Lijiang Yunxin Green Biological Development Co., Ltd., in NW Yunnan Province, China, where located in Three Parallel Rivers and one of hotpot on biodiversity conservation in the world, in May 2020 and identified based on morphological features by Professor Heng Li (CAS Key Laboratory for Plant Diversity and Biogeography of East Asia, Kunming Institute of Botany, Chinese Academy of Sciences, China).

### 2.4 Extraction and isolation

The dried rhizomes of *P. polyphylla* var. *yunnanensis* (2.1 kg) were crushed into powder with a pulverizer and extracted in 80% (v/v) ethanol (3 × 6 L) with an ultrasonicator (40 KHZ) at 60°C for 2 h. The ethanol extract was filtered by filter paper, and the resulting filtrate was concentrated as crude extract (A, 400 g). A 300-g aliquot of the crude extract was separated on a macroporous resin column with four concentrations of ethanol to obtain an aqueous fraction (**A**_**1**_, 170 g), a 50% ethanol fraction (**A**_**2**_, 23 g), a 80% ethanol fraction (**A**_**3**_, 5 g), and a 95% ethanol fraction (**A**_**4**_, 4 g) (Supplementary Figure S7, in the Supplementary Material S2).

Fraction A3 (2.1 g) was isolated by column chromatography (silica gel, 200–300 mesh, CH_2_Cl_2_/CH_3_OH, 19:1→0:1, v/v) as fractions A3–1 (186.3 mg), A3–2 (498.6 mg), A3–3 (300.1 mg), and A3–4 (103.2 mg). An aliquot from fraction A3–2 (109 mg) was separated by semi-preparative HPLC (Zorbax SB-C18 column, 9.4 × 250 mm; CH_3_CN/0.1% [v/v] H_3_PO_4_ solution, 1 mL/min, 30°C, CH_3_CN, 0–15 min, 0%–20%; 15–20 min, 20%–35%; 20–25 min, 35%–55%; 25–35 min, 55%–60%; 35–40 min, 60%–75%; 40–45 min, 70%–100%; 45–55 min, 100%–100%) to obtain four saponins: Paris saponin Pb (**1**, 12.9 mg, pennogenin-3-O-α-L-rhamnopyranosyl- (1→2)-[α-L-rhamnopyranosyl-(1→4)]-β-D-glucopyranoside, t_R_ = 35.05 min), Paris saponin H (**2**, 11.1 mg, pennogenin-3-O-α-L-arabinofuranosyl-(1→4)-[α-L-rhamnopyranosyl-(1→2)]-β-D-glycopyranoside, t_R_ = 35.76 min), Paris saponin III (**3**, 23.3 mg, diosgenin-3-O-α-L-rhamnopyranosyl-(1→2)-[α-L-rhamnopyranosyl-(1→ 4)]-β-D-glucopyranoside, t_R_ = 42.68 min), and Paris saponin I (**4**, 26.0 mg, diosgenin-3-O-α-L-arabinofuranosyl-(1→4)-[α-L-rhamnopyranosyl-(1→2)]-β-D-glucopyranoside, t_R_ = 44.24 min). An aliquot of fraction A3–3 (40.1 mg) was similarly separated by preparative HPLC (Zorbax SB-C18 column, 9.4 × 250 mm; CH_3_CN/H_2_O, 3 mL/min, 30°C, CH_3_CN, 0–20 min, 30%–60%; 20–25 min, 60%–30%) to obtain two saponins: Paris saponin VII (**5**, 15.2 mg, pennogenin 3-O-α-L-rhamnopyranosyl-(1→2)-[α-L-rhamnopyranosyl-(1→4)-α-L-rhamnopyranosyl-(1→4)]-β-D-glucopyranoside, t_R_ = 18.26 min) and Paris saponin II (**6**, 7.6 mg, diosgenin-3-O-α-L-rhamnopyranosyl-(1→2)-[α-L-rhamnopyranosyl-(1→4)-α-L-rhamnopyranosyl-(1→4)]-β-D-glucopyranoside, t_R_ = 22.01 min) (Supplementary Figure S8, in the Supplementary Material S2).

### 2.5 Analysis and determination of total saponin content

The contents of total saponins in dry rhizomes were determined by the method described in the Chinese Pharmacopoeia (National Pharmacopoeia Committee, 2020). The dry rhizomes were crushed into powder and passed through a 50 mesh sieve (0.28 mm). The powder (498.8 mg) was placed in a conical flask with a cover, to which 25 mL ethanol was added before the weight of the system was measured. The system was refluxed for 30 min and cooled to room temperature. The weight of the entire system was determined again and brought to its original weight with ethanol. Five extracts (A, A_1_, A_2_, A_3_, and A_4_) in the analysis and determination of total saponin were prepared at concentrations of 9.45, 4.45, 11.50, 10.90, and 12.75 mg/mL in methanol, respectively. The liquid was filtered through a 0.45-μm membrane before use for HPLC analysis.

Samples were analyzed by HPLC (Agilent 1260 series system, Zorbax SB-C18 column, 5 μm, ϕ 4.6 × 250 mm), with a mobile phase consisting of CH_3_CN/H_2_O, with a flow rate of 1 mL/min, at 30°C, starting with CH_3_CN, 0–20 min, followed by 30%–60% CH_3_CN (in water, v/v); 20–25 min, 60%–30%. The injection volumes were 10 μL for the dry rhizome powder and 5 μL for each of the six extracts. Eight standard saponins (Paris saponins I, II, VII, III, V, VI, Pb, and H) were purchased from Sichuan Weikeqi Biological Technology Co., Ltd. (purity ≥98%).

### 2.6 Human cell lines and culture

Three human liver cancer cell lines (SMMC-7721, HepG2, and SK-HEP-1) and the non-small-cell lung cancer (NSCLC) cell line A549 were obtained from ATCC (Manassas, VA, United States). Normal human cell lines (liver cell line LO2 and lung cell line BEAS-2B) were obtained from ATCC (Manassas, VA, United States). Cells were cultured in RMPI-1640 or DMEM medium (Biological Industries, Kibbutz Beit-Haemek, Israel) supplemented with 10% (v/v) fetal bovine serum (Biological Industries) at 37°C in a humidified atmosphere with 5% (v/v) CO_2_.

### 2.7 Cell viability assays

The effects of the extracts and isolated saponins on cell growth were evaluated by the MTS assay (3-(4,5-dimethylthiazol-2-yl)-5(3-carboxymethoxyphenyl)-2-(4-sulfopheny)-2H-tetrazolium; Promega, Madison, WI, United States) on human tumor cell lines (Mosmann, 1983; Cory et al., 1991; Malich et al., 1997). In a test experiment of cell inhibition, the cell inhibition effects of six crude extracts (100 μg/mL) and six isolated saponins (40 μM) were roughly measured to ascertain whether to measure the IC_50_ value by MTS assay.

Each well of a 96-well plate (Multiskan Fc, Therm) was seeded with 100 μL cells at a density of 3,000–5,000 cells per well, with the cell suspension containing 10% (v/v) fetal bovine serum (DMEM or RMPI1640, Biological Industries, Kibbutz Beit-Haemek, Israel). After 12–24 h of inoculation and culture at 37°C, the extracts or saponins were added at the indicated concentrations after being dissolved in dimethyl sulfoxide (DMSO, Biological Industries, Kibbutz Beit-Haemek, Israel) in a final volume of 200 μL per well, each in triplicate. The six crude extracts were added at concentrations of 100, 20, 4, 0.8, and 0.16 μg/mL; the six isolated saponins were added at concentrations of 10, 5, 2.5, 1.25, 0.625, and 0.3125 μM. After culture for 48 h at 37°C, 20 μL of MTS solution and 100 μL of DMEM were added to each well; three blank wells were set aside with 20 μL MTS solution and 100 μL DMEM. After an incubation for another 2–4 h, the absorbance at 492 nm was measured. Cisplatin and Paclitaxel (MeilunBio) were used as a positive control in each experiment. The cell growth curve was drawn with the concentration as the abscissa and the cell survival rate as the ordinate. The IC_50_ value of each saponin was calculated by the two-point method (Reed and Muench, 1938).

### 2.8 Cell cycle analysis

Cell cycle distribution was determined by propidium iodide (PI, BD, Pharmingen) staining (Kong et al., 2016; Wei et al., 2021). Briefly, three cancer cell lines were cultured in six-well plates at a density of 3 × 10^5^ cells per well for 24 h before treatment with each of the six isolated saponins at the indicated concentrations for 24 h. The cells were collected, centrifuged, and washed twice with ice-cold PBS. Cells were fixed in 70% (v/v) ice-cold ethanol at 4°C overnight and washed again in PBS twice. The cells were incubated with 100 μL RNase A at 37°C for 30 min in the dark, after which 400 μL PI was added to the cells with RNase A in the dark at 4°C for 15 min. The cell cycle stage was analyzed by flow cytometry (BD, FACSCelesta, United States) with FLOWJO software (FlowJo, Ashland, OR, United States).

### 2.9 Determination of mitochondrial membrane potential

The probe 5,6-dichloro-1,1′,3,3′- tetraethyl-imidacarbocyanine iodide (JC-10) was used as a measure of mitochondrial membrane potential (Hirose et al., 1974; Chaudhari et al., 2008). The effects of each of the six isolated saponins on mitochondrial membrane potential were determined using a JC-10 assay kit (×200, 10 μL/tube, five tubes), which was purchased from Beijing Solarbio Science & Technology Co., Ltd. (Beijing, China). Cells were cultured in a confocal dish with this method. After 12–24 h of inoculation and culture at 37°C, the purified saponins were added at the indicated concentrations. The cells were incubated at 37°C in a humidified atmosphere with 5% (v/v) CO_2_ for 24 h. The culture medium was removed, and the confocal dishes were washed twice with 1 mL PBS, followed by 0.5 mL incubation buffer containing 2.5 μL JC-10 (×200) at 37°C for 20 min in the dark. After incubation, the supernatant was removed, and the cells were washed twice with 1 mL JC-10 buffer and PBS. PBS (1 mL) was added to each well of the confocal dish, and the stained cells were observed with a confocal laser scanning microscope (TCS SP8X, Leica, Germany) using a ×40 objective. The wavelengths for excitation were 490 nm (green fluorescence) and 525 nm (red fluorescence). The wavelengths for emission were in the range of 495–565 nm (monomers for JC-10) and 550–620 nm (polymer for JC-10).

### 2.10 Apoptosis determination by annexin V-propidium iodide staining

Double staining with annexin V-fluorescein isothiocyanate (FITC) and PI was used to determine the effects of the six saponins on cell apoptosis by flow cytometry (Homburg et al., 1995; Vermes et al., 1995; Casciola-Rosen et al., 1996). The effects of the six isolated saponins on apoptosis were determined using an annexin V-FITC apoptosis kit (BD, Pharmingen); the chemotherapy drug doxorubicin (DOX, Beyotime Institute of Biotechnology, Shanghai, China) was used as the positive control. Cells were cultured in six-well plates at a density of 3 × 10^5^ cells per well for 24 h before being treated with each of the six isolated saponins at the indicated concentrations for 24 h. The cells were collected, centrifuged, and washed twice with ice-cold phosphate-buffered saline (PBS). The cells were stained with annexin V-FITC and PI for 15 min in the dark at room temperature before analysis by flow cytometry (BD, FACSCelesta, America). Annexin V-FITC^+^ and PI^+^ (Q2) were considered late-stage apoptotic cells, while annexin V-FITC^+^ and PI^−^ (Q3) cells were considered early-stage apoptotic cells. Annexin V-FITC^–^ and PI^+^ (Q1) were necrotic or mechanically damaged cells, and annexin V-FITC^–^ and PI^−^ (Q4) cells were healthy living cells.

## 3 Results and discussion

### 3.1 Isolation and identification of substances based on indigenous medicine knowledge

**FIGURE 1:**
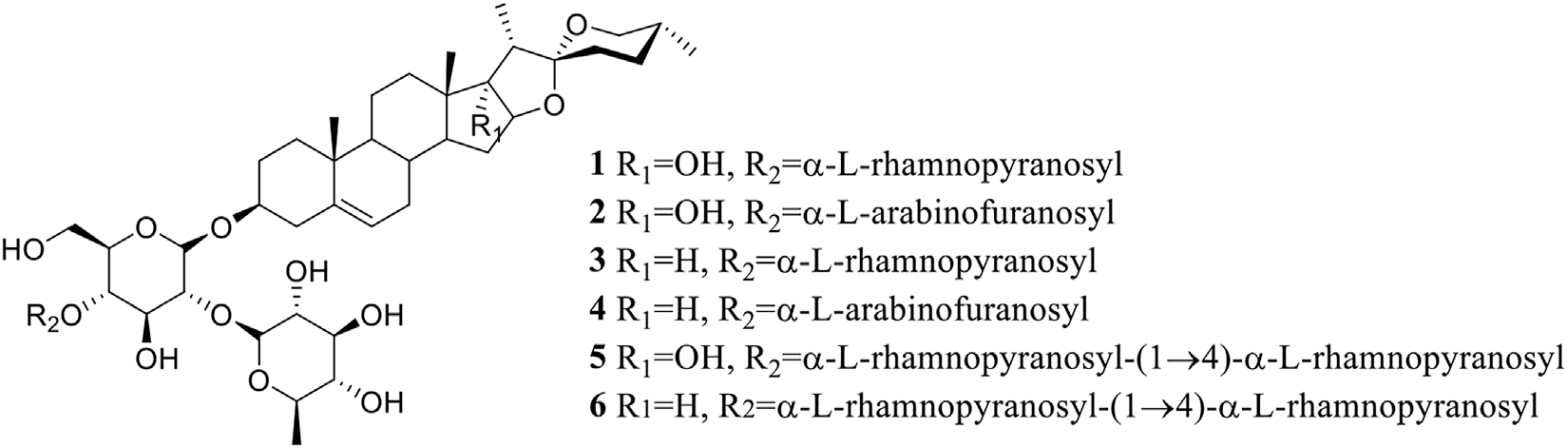
Structure of saponins **1**–**6** extracted from *P. polyphylla* var. *yunnanensis*.

The saponins **1**–**6** (Figure 1) were isolated from the dried rhizomes of *P. polyphylla* var. *yunnanensis* and were identified to be Paris saponin Pb (**1**, pennogenin-3-O-α-L-rhamnopyranosyl-(1→2)-[α-L-rhamnopyranosyl-(1→4)]-β-D-glucopyranoside) (Nakano et al., 1989; Hua et al., 2015), Paris saponin H (**2**, pennogenin-3-O-α-L-arabinofuranosyl-(1→4)-[α-L-rhamnopyranosyl-(1→2)]-β-D-glycopyranoside) (Chen and Zhou, 1987; Wang et al., 2007; Pang et al., 2015), Paris saponin III (**3**, diosgenin-3-O-α-L-rhamnopyranosyl-(1→2)-[α-L-rhamnopyranosyl- (1→4)]-β-D-glucopyranoside) (Han et al., 1999; Wang et al., 2001; Wang et al., 2007), Paris saponin I (**4**, diosgenin-3-O-α-L-arabinofuranosyl-(1→4)-[α-L-rhamnopyranosyl-(1→2)]-β-D-glucopyranoside) (Chen and Zhou, 1987; Huang et al., 2009; Jing et al., 2017), Paris saponin VII (**5**, pennogenin 3-O-α-L-rhamnopyranosyl-(1→2)-[α-L-rhamnopyranosyl-(1→4)-α-L-rhamnopyranosyl-(1→4)]-β-D-glucopyranoside) (Chen et al., 1983; Zhang et al., 2014), and Paris saponin II (**6**, diosgenin-3-O-α-L-rhamnopyranosyl-(1→2)-[α-L-rhamnopyranosyl-(1→4)-α-L-rhamnopyranosyl-(1→4)]-β-D-glucopyranoside) (Munday et al., 1993; Jing et al., 2017) by their nuclear magnetic resonance (NMR) and mass spectrometry (MS) data, which were provided in the Supplementary Material. These values were all consistent with the known saponins in the reported literature.

### 3.2 High-performance liquid chromatography analysis of total saponin contents

The Chinese Pharmacopoeia details a method for the determination of total saponin contents in *P. polyphylla* extracts (National Pharmacopoeia Committee, 2020), whose levels in dry TCM powder should reach at least 0.6% (National Pharmacopoeia Committee, 2020) to be effective and should include Paris saponin I (C_44_H_70_O_16_), Paris saponin II (C_51_H_82_O_20_), and Paris saponin VII (C_51_H_82_O_21_). Accordingly, total saponin contents in the powder of dry rhizomes and six ethanol or aqueous extracts were measured using eight saponins as standards (Table 1). Six saponins (Paris saponins I, II, VII, III, H, and Pb) were detected in dry rhizome powder; importantly, the contents for Paris saponins I, II, and VII were much higher (6.96%) than the contents set by the Chinese Pharmacopoeia. Paris saponins I, II, and VII and total saponins reached their highest contents in fraction A_3_, which was obtained by elution from the macroporous resin column with 80% ethanol. These two sets of saponins comprised 37.06% (Paris saponins I, II, and VII) and 59.38% (total saponins) of this fraction. Therefore, fraction A_3_ was subjected to further separation for the purification of the six detected saponins.

**TABLE 1:** Analysis and determination of total saponin contents in dried rhizome powder and six ethanol extracts.

No.	Saponins detected	Contents	
		Paris saponins I, II, and VII (%)	Total Paris saponins (%)
Powder of dried rhizomes	Paris saponins I, II, VII, III, H, and Pb	6.96	10.51
A	Paris saponins I, II, VII, III, and H	2.79	4.11
A_1_	Paris saponin III	—	0.47
A_2_	Paris saponins I, II, VII, III, H, and Pb	3.90	6.03
A_3_	Paris saponins I, II, VII, III, V, VI, H, and Pb	37.06	59.38
A_4_	Paris saponins I, II, VII, III, V, and H	6.70	10.96

Note: —, not detected.

Steroidal saponins accumulate to different levels in *Paris* plants each year and as a function of their natural habitat (Wang et al., 2015). In addition, distinct *Paris* species present different steroidal saponin profiles (Wei et al., 2014); for example, 112 steroidal saponins were detected in *P. polyphylla* var. *yunnanensis* (Zhou et al., 2022). The saponin contents of the *P. polyphylla* var. *yunnanensis* rhizomes tested here were much higher than those set by the standards of the Chinese Pharmacopoeia in 2020, which used plants grown in Lijiang, Yunnan province, China. The samples analyzed here mainly contained the eight Paris saponins I, II, VII, III, V, VI, H, and Pb (Table 1).

### 3.3 Evaluation of cytotoxicity activity

The growth inhibitory activity of all extracts and saponins were obtained from the dried *P. polyphylla* var. *yunnanensis* rhizomes. A preliminary experiment indicated that five crude extracts (A, A_1_, A_2_, A_3_, and A_4_) inhibit cell division when applied at 100 μg/mL in the pre-experiment. To facilitate comparisons, the IC_50_ values of five extracts (A, A_1_, A_2_, A_3_, and A_4_) were determined in the three human liver cancer cell lines SMMC-7721, HepG2, and SK-HEP-1 as well as in the NSCLC cell line A549 by the MTS method (Table 2). Among all crude extracts, A_3_ was the most active against all four human cancer cell lines tested, with IC_50_ values of 1.226 μg/mL (SMMC-7721), 1.878 μg/mL (HepG2), 0.878 μg/mL (SK-HEP-1), and 1.287 μg/mL (A549).

**TABLE 2:** IC_50_ values for crude extracts and saponins (1–6) from *P. polyphylla* var. *yunnanensis* against four human cancer cell lines.

No.	Extracts and saponins	IC_50_ (±SD)			
		Liver cancer cell line			NSCLC
		SMMC-7721	HepG2	SK-HEP-1	A549
A	Crude extracts (μg/mL)	9.82 ± 0.23	33.31 ± 1.51	10.53 ± 0.27	13.78 ± 0.20
A_1_		27.50 ± 0.57	53.27 ± 1.76	23.08 ± 0.72	28.81 ± 0.67
A_2_		2.09 ± 0.10	3.08 ± 0.09	6.41 ± 0.19	1.64 ± 0.095
A_3_		1.23 ± 0.06	1.88 ± 0.05	0.88 ± 0.03	1.29 ± 0.04
A_4_		7.57 ± 0.27	10.70 ± 0.18	13.75 ± 0.83	7.99 ± 0.15
Cisplatin	Positive control (μg/mL)	5.05 ± 0.12	1.90 ± 0.13	8.79 ± 0.49	16.74 ± 0.68
Paclitaxel		0.12 ± 0.02	<0.01	<0.01	<0.01
**1**	Saponins (μM)	0.76 ± 0.03	1.78 ± 0.01	0.90 ± 0.04	0.98 ± 0.01
**2**		1.88 ± 0.07	2.14 ± 0.01	1.58 ± 0.08	1.25 ± 0.02
**3**		2.56 ± 0.08	3.80 ± 0.05	1.80 ± 0.07	1.69 ± 0.04
**4**		0.72 ± 0.02	1.59 ± 0.03	0.66 ± 0.04	0.58 ± 0.01
**5**		1.20 ± 0.10	1.57 ± 0.01	0.94 ± 0.06	0.90 ± 0.01
**6**		0.45 ± 0.01	2.59 ± 0.03	1.30 ± 0.06	0.24 ± 0.01
Cisplatin	Positive control (μM)	17.83 ± 0.57	5.67 ± 0.10	21.02 ± 0.71	26.08 ± 0.52
Paclitaxel		0.18 ± 0.01	<0.01	<0.01	<0.01

Six main saponins (**1**–**6**) were obtained from the further isolated and identified in the A_3_ fraction (Figure 1), and their IC_50_ values for growth inhibition were determined using the same four human cancer cell lines. Importantly, all IC_50_ values were lower than those obtained with the positive control cisplatin, a commonly used chemotherapy drug (Table 2). In addition, the normal liver and lung cells (LO2 and BEAS-2B) had also been evaluated for six saponins (Table 3). However, none of the purified saponins exhibited the same level of activity as the other chemotherapy drug paclitaxel. Moreover, each saponin showed a distinct activity when tested against the four cancer cell lines, with no single saponin being most effective against all cell lines. More specifically, Paris saponin II (**6**) was most active against NSCLC, as evidenced by the low IC_50_ values. Likewise, Paris saponins I (**4**) and Pb (**1**) were most active against the three liver cancer cell lines. A previous review mentioned that toxicity evaluation suggested that Rhizome Paridis had slight liver toxicity (Ding et al., 2021). However, the present data for normal cells supported this view (Table 3).

**TABLE 3:** IC_50_ values for saponins (1–6) from *P. polyphylla* var. *yunnanensis* against normal liver and lung cell lines.

No.	Saponins	IC_50_ (±SD)	
		Normal liver cell	Normal lung cell
		LO2	BEAS-2B
**1**	Saponins (μM)	1.79 ± 0.10	1.19 ± 0.01
**2**		2.33 ± 0.10	4.95 ± 0.04
**3**		3.78 ± 0.18	3.07 ± 0.19
**4**		0.85 ± 0.01	1.82 ± 0.09
**5**		0.90 ± 0.02	0.94 ± 0.03
**6**		0.37 ± 0.01	0.73 ± 0.03
Cisplatin	Positive control (μM)	13.78 ± 0.48	>40
Paclitaxel		<0.01	>5

Paris saponins can be divided into diosgenin-type (saponins **3**, **4**, and **6**) and pennogenin-type (saponins **1**, **2**, and **5**) as a function of the presence of a hydroxyl group at site 17 (chain R1 in Figure 1) (Man et al., 2013; Wang et al., 2018). However, the results showed that the activities of these two kinds of saponins provided limitation, related different results compared with pre-evidence (Table 2). Although, the species of *Paris* has been extensively used for cancer therapy in indigenous medicine knowledge (Yan et al., 2021), research on the anticancer properties and potential mechanisms is still a preliminary exploration mainly due to the limitation of their low in plants. With the development of artificial cultivation techniques of *Paris* species for the past few years, more and more studies of metabolites and pharmacology are being performed to further identify effective steroidal saponins as potential anticancer drugs (Long et al., 2015; Qin et al., 2018). The results of MTS assay further confirmed the correlation between total saponin content in crude extracts and anticarcinogenic activity. What’s more, the difference in the anticancer activity of six saponins should be highly associated with the type, binding mode and number of glycosyl (Figure 1; Tables 1, 2).

### 3.4 Paris saponins induce cell cycle arrest in cancer cell lines

**FIGURE 2:**
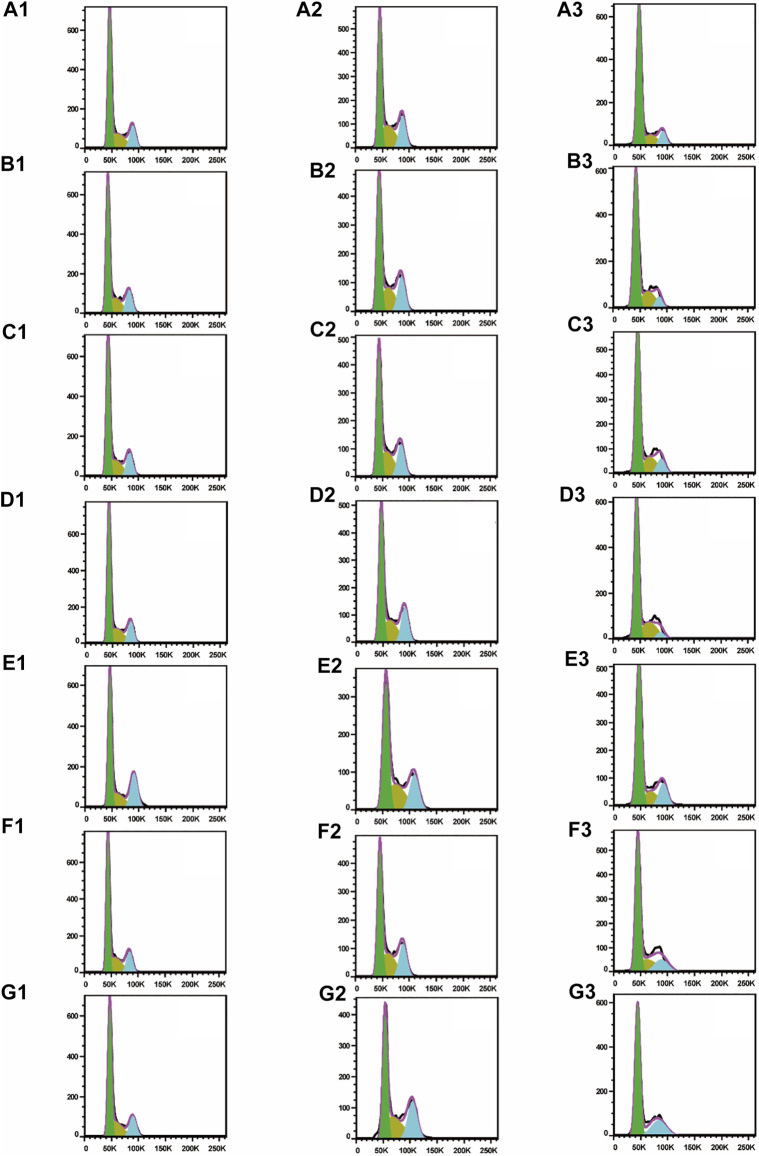
All six isolated saponins induce cell cycle arrest in three cancer cell lines. (A1–G1), A549 cells. (A2–G2), SMMC-7721 cells. (A3–G3), HepG2 cells. DMSO was used as a negative control (An). Saponin **1** (Bn), saponin **2** (Cn), saponin **3** (Dn), saponin **4** (En), saponin **5** (Fn), and saponin **6** (Gn), n = 1, 2, 3. For SMMC-7721 and HepG2 cell lines, each saponin was added at 1 μM. For A549 cells, saponins **1** and **5** were added at 0.5 μM; the other four saponins were added at 1 μM.

**FIGURE 3:**
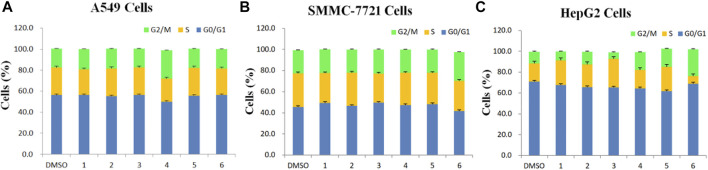
Determination of cell cycle stage in three cancer cell lines (A549, SMMC-7721 and HepG2) in the presence of purified saponins.

To test whether inhibition of the growth of three human cancer cells also affected cell cycle progression (G0/G1, S, and G2/M), induction of A549, SMMC-7721, and HepG2 cell cycles was detected by propidium iodide (PI) staining and flow cytometry after six isolated saponin treatments (Figures 2Bn–Gn). The same cell lines with DMSO were only treated as a negative control (Figure 2An). Substantial changes were detected in the cell cycle progression of the three human cancer cell lines when treated with Paris saponins (Figures 2, 3).

Compared to the DMSO control, saponin **2** increased the proportion of A549 cells in the S and G2/M phases, while saponins **1**, **4**, and **6** increased the proportion of cells in the G2/M phase (Figures 2A1–G1). Saponin **5** increased the proportion of cells in S phase, and saponin **3** increased the proportion of cells in the G0/G1 phase.

The six Paris saponins also induced cell cycle arrest in the SMMC-7721 cell line (Figures 2A2–G2). Compared to the DMSO control, saponin **6** increased the proportion of cells in the G2/M phase by 27.10%, while the other five saponins increased the proportion of cells in the G0/G1 phase. The HepG2 cell line was also sensitive to Paris saponins (Figures 2A3–G3). Compared to the DMSO control, saponins **4** and **6** increased the proportion of cells in the G2/M phase by 17.2% and 25.8%, respectively; saponins **1** and **3** increased the proportion of cells in S phase by 23.7% and 27.5%, respectively; and saponins **2** and **5** increased the proportion of cells in the S and G2/M phases.

Many anticancer medications inhibit the growth of tumor cells by targeting the S and G2/M phases of the cell cycle in cancer cells. Indeed, both S and G2/M phases are very important for the proliferation of malignant cancer cells (Sui et al., 2016). A closer look at the flow cytometry data revealed that the proportion of HepG2 cells at the S or G2/M phase treated with any one of the six saponins is higher relative to the DMSO control, suggesting that all six saponins block the cell cycle at the S or G2/M phases (Figure 3C). For the other liver cancer cell line, SMMC-7721, only saponin **6** caused cell cycle arrest at the G2/M phase (Figure 3B). For the A549 lung cancer cell line, only saponin **4** showed a significant effect on the relative proportions of cells in each cell cycle stage, with an increase in the proportion of cells at the G2/M phase, suggestive of cell cycle arrest (Figure 3A).

These results indicate that the six Paris saponins inhibit cancer cell growth by inducing cell cycle arrest at the S or G2/M phase.

### 3.5 Paris saponins cause a loss of mitochondrial membrane potential

**FIGURE 4:**
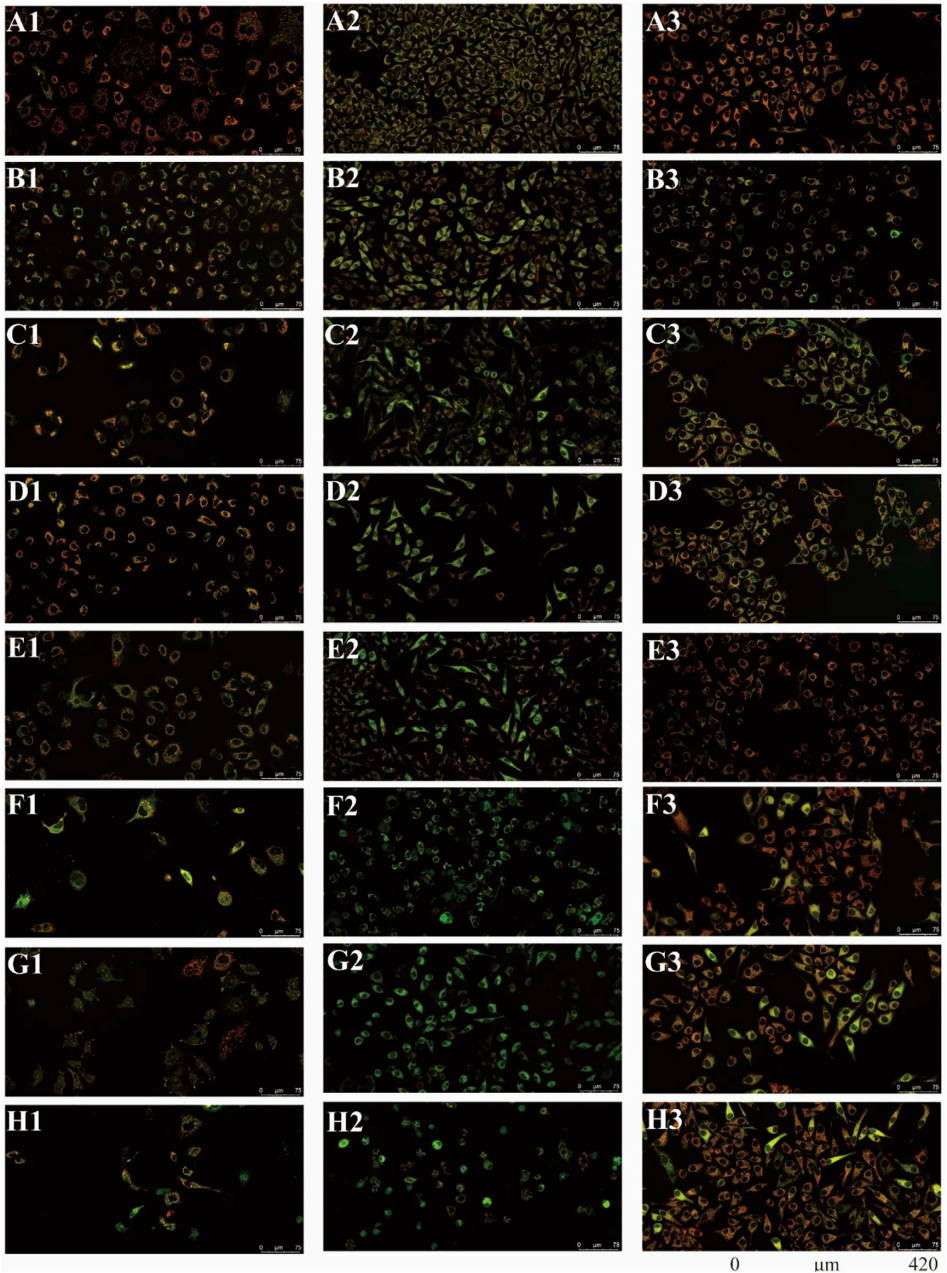
All six isolated saponins affect the mitochondrial membrane potential of three cancer cell lines. (A1–H1), A549 cells. (A2–H2), SMMC-7721 cells. (A3–H3), HepG2 cells. Untreated groups served as negative control (An). CCCP-treated groups served as a positive control at 2 μM (Bn). All saponins were added at a concentration of 2 μM: saponin **1** (Cn), saponin **2** (Dn), saponin **3** (En), saponin **4** (Fn), saponin **5** (Gn), and saponin **6** (Hn), n = 1, 2, 3.

**FIGURE 5:**
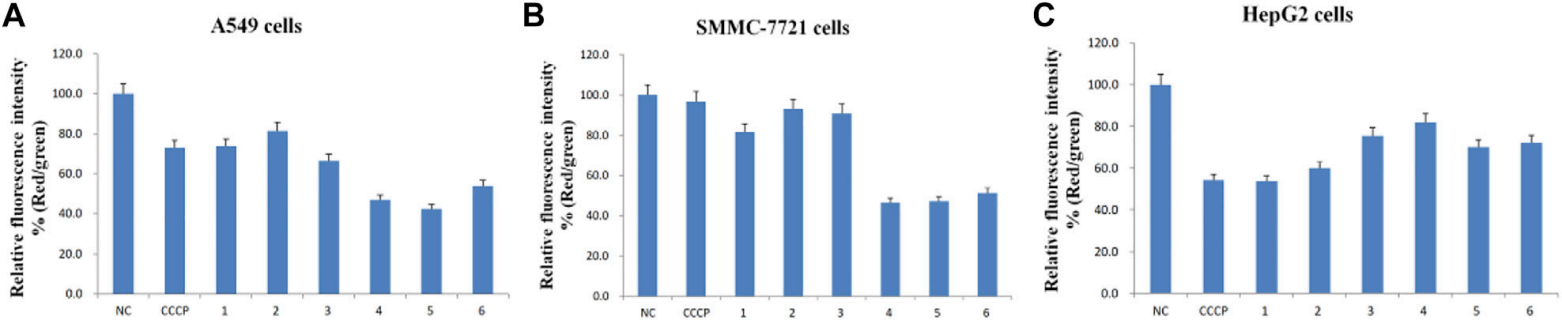
Relative fluorescence intensity (Red/green) of the six purified saponins in three cancer cell lines (A549, SMMC-7721 and HepG2).

There were two main apoptotic pathways in tumor cells, including the cell surface death receptor-mediated pathway and the mitochondrial apoptosis pathway, the latter being at the core of apoptosis induction (Sui et al., 2016). The six isolated saponins were therefore assessed for their ability to induce early apoptotic cell death in three human cancer cell lines (A549, SMMC-7721, and HepG2) using a mitochondrial membrane potential assay kit based on the potential-dependent accumulation of the JC-10 dye inside mitochondria (Figures 4, 5; Supplementary Figures S9–S11). Red fluorescence from the JC-10 probe indicates a normal mitochondrial membrane potential, while a shift to green fluorescence reflects a drop in membrane potential, which is suggestive of a cell in the early stage of apoptosis.

Cancer cells showed red or yellow-green fluorescence in the absence of any treatment, indicating that their mitochondrial membrane potential is normal (Figures 4A1–A3), which used as negative control (NC). As a positive control for cells with depolarized mitochondrial membranes, cells were treated with the proton ion mitochondrial uncoupling agent carbonyl cyanide 3-chlorophenylhydrazone (CCCP), which resulted in strong green fluorescence (Figures 4B1–B3). Each cancer cell line was also treated with one of the six isolated saponins (Figures 4Cn–Hn). However, the merge by red and green fluorescence for three human cancer cells of six saponins were showed in Figure 4. Also, the merge, green channel and red channel for each human cancer cells of six saponins were showed in Supplementary Figures S9–S11 in the Supplementary Material S2, respectively. Compared to the NC and CCCP control, the green fluorescence was generally higher than red fluorescence in SMMC-7721 cells, next is A549 cells. And for HepG2 cells, the red fluorescence was higher than green fluorescence (Figure 4). Importantly, all purified saponins displayed an increase in green fluorescence over the untreated controls. This finding indicated that the six saponins purified from dried *P. polyphylla* var. *yunnanensis* rhizomes increase the permeability of the mitochondrion membrane, leading to a decrease in mitochondrial membrane potential.

JC-10 fluorescent probe can enter mitochondria in normal cells when the membrane potential of mitochondria is high, and it exists in the form of polymer, showing red fluorescence. During the period of cell apoptosis, the mitochondrial membrane potential decreases, and JC-10 is dissociated in the cytoplasm in the form of monomer, showing green fluorescence (Hirose et al., 1974; Castedo et al., 2002). In this study, the ratio of relative fluorescence intensity was used to evaluate the changes of mitochondrial membrane potential, which was indicated by the ratio of red/green fluorescence (Figure 5). The lower the ratio of relative fluorescence intensity was, the more red fluorescence and the less green fluorescence were, which was indicated more severe the damage to the cell membrane, resulting in lower mitochondrial membrane potential. As shown in Figure 5, the ratio of relative fluorescence intensity was decreased in different levels after treating with six saponins, which indicated that the six saponins caused the decrease of mitochondrial membrane potential by damaging mitochondrial membrane, and suggested that cancer cells were in the early stage of cell apoptosis.

To sum up, six Paris saponins may increase the permeability of the mitochondrion membrane in different levels, leading to a decrease in mitochondrial membrane potential.

### 3.6 Paris saponins induce apoptosis in cancer cell lines

**FIGURE 6:**
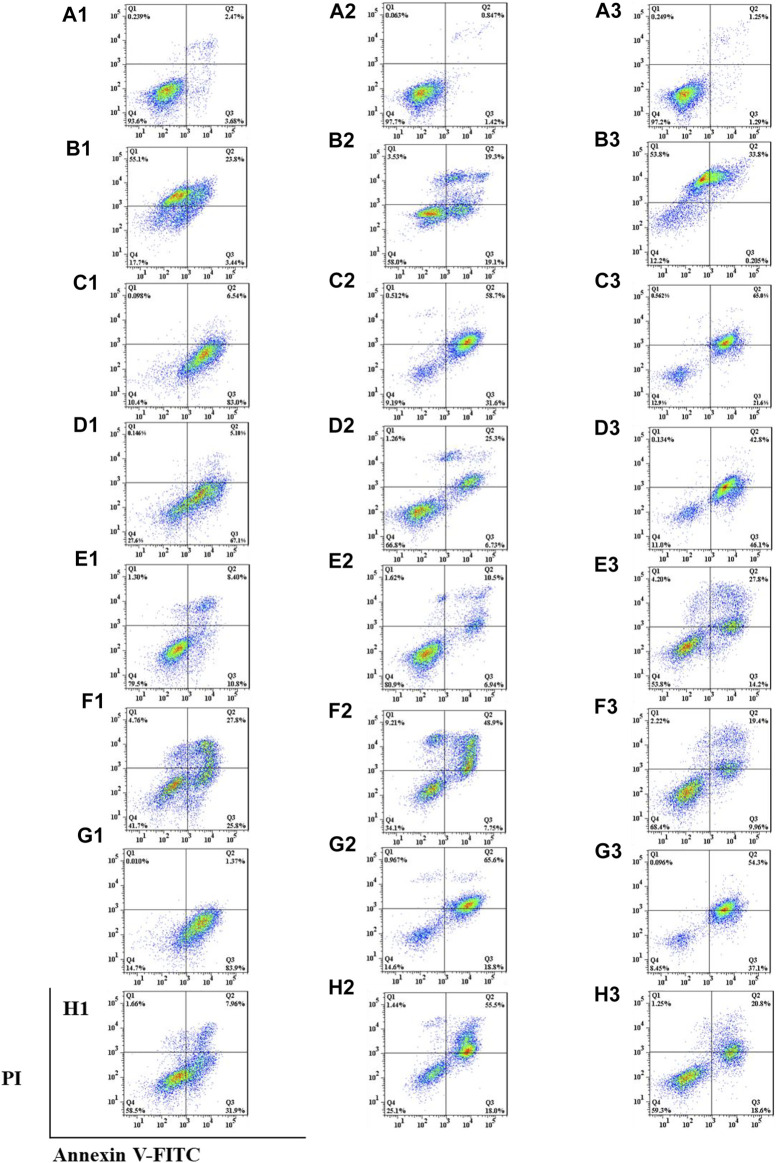
All six isolated saponins induce apoptosis in three cancer cell lines. (A1–H1), A549 cells. (A2–H2), SMMC-7721 cells. (A3–H3), HepG2 cells. As negative control, 0.1% (v/v) DMSO was used (An). DOX-treated cultures served as positive control at concentrations of 5, 1, and 5 μM (Bn). All saponins were added at a concentration of 5 μM: saponin **1** (Cn), saponin **2** (Dn), saponin **3** (En), saponin **4** (Fn), saponin **5** (Gn), and saponin **6** (Hn), n=1, 2, 3.

Anticancer drugs inhibit the proliferation of tumor cells by increasing the apoptosis rate (Foo et al., 2015). Encouraged by the results from the mitochondrial membrane potential assay above, the effects of the six isolated saponins on apoptosis were determined by double staining with annexin V-FITC and PI in three human cancer cell lines, including A549, SMMC-7721, and HepG2 (Figure 6). Cancer cells were treated with 0.1% DMSO as a negative control (Figure 6An) and the chemotherapy drug doxorubicin (DOX) as a positive control (Figure 6Bn). These six isolated saponins significantly increased early-stage and late-stage apoptotic cells, as reflected by the proportion of annexin V-FITC^+^/PI^−^ and annexin V-FITC^+^/PI^+^ cells, respectively (Figures 6Cn–Hn). A concentration of 5 μM for each saponin was sufficient to promote apoptosis in the three cancel cell lines tested here. The effects of six saponins on promoting apoptosis were equivalent to that of DOX, or even more than that of DOX.

**FIGURE 7:**
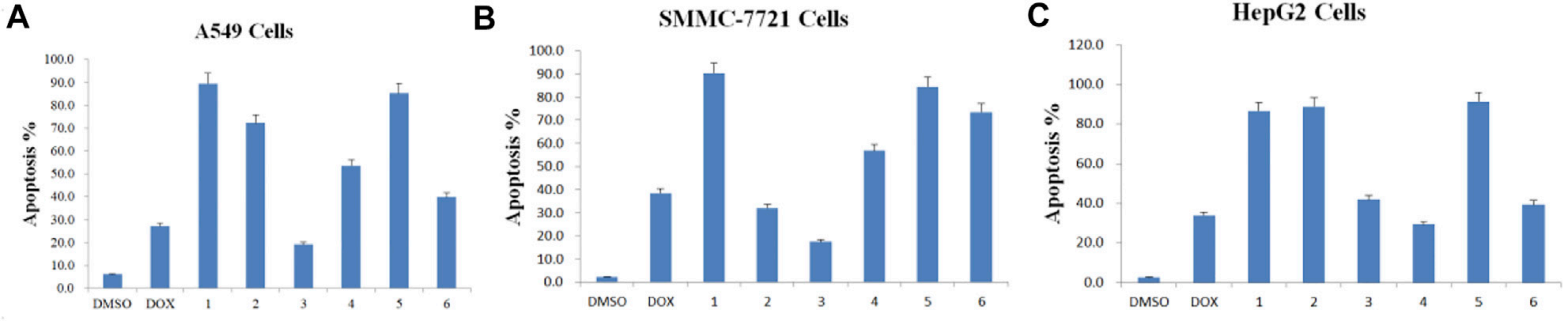
Apoptosis rate induced by each of the six purified saponins in three cancer cell lines (A549, SMMC-7721 and HepG2).

Apoptosis rate of each cell line was calculated in the absence or presence of each purified Paris saponin (Figure 7). Saponin **1** was the most effective in inducing apoptosis in A549 and SMMC-7721 cells and was also effective in HepG2 cells. Saponins **2** and **5** also induced strong apoptosis in A549 cells, while saponins **5** and **6** exhibited a substantial induction of apoptosis in SMMC-7721 cells. For HepG2 cells, the highest apoptosis rate was saponin **5**, and next were saponins **2** and **1**. Notably, saponins **5** and **1** consistently induced stronger apoptosis than the positive control DOX in all three cancer cell lines. In conclusion, six saponins induced significant apoptosis in the three human cancer cell lines, supporting their potential as inhibitory saponins against the proliferation of cancer cells.

We found six saponins inhibited cancer cell growth in three human cancer cells was further demonstrated (Table 2). Moreover, the anticancer activity of six saponins were associated with induction of early- and late-stage apoptosis and cell cycle G2/M or S phase arrest in there human cancer cells (Figures 2, 6). Apoptosis, or programmed cell death, plays a crucial role in controlling cell number in many developmental and physiological settings and in chemotherapy-induced tumour-cell killing, through regulating the normal balance between cell death and survival (Hu and Kavanagh, 2003). Therefore, apoptosis inducers of cancer cells have therapeutic potential in chemoprevention and chemotherapy of cancer (Long et al., 2015). The study showed that the breakdown of mitochondrial membrane is able to enhance the permeability of outer mitochondrial membrane. The release of induced cytochrome and other apoptotic factors from mitochondria into the cytosol is critical to activate the intrinsic apoptotic pathway (Long et al., 2015). Herein, the main effects of six saponins in mitochondrial apoptosis pathway were detected by JC-10 staining and Annexin V- FITC & PI staining in human cancer cells, respectively (Figures 4, 6). Our findings suggest that anti-cancer activity of six saponins is associated with apoptotic induction through a decrease in mitochondrial membrane potential in A549, SMMC-7721, and HepG2 cells.

## 4 Conclusion

An ethnobotanical study of the indigenous people and local uses of *P. polyphylla* var. *yunnanensis* is established, which revealed four ethnic groups historical practice from the rhizome of this plant to treat tumors, raising the possibility that *P. polyphylla* var. *yunnanensis* may contain potential anticancer metabolites (Yan et al., 2021). Therefore, it is worth to verify the anticancer activity of indigenous and local use for cultivated *P. polyphylla* var. *yunnanensis.* Based on the results of ethnobotanical study, an interdisciplinary approach has been used to study active metabolites in this cultivated species. Five crude extracts were obtained while determined their total saponin contents and cytotoxicity activity. The fraction A_3_ is the highest of five parts for total saponin contents and cytotoxicity activity. Six Paris saponins from the fraction A_3_ were isolated, purified and their potential pro-apoptotic activity for use as anti-proliferative drugs was tested. Cultivated *P. polyphylla* var. *yunnanensis* rhizomes are a preferable source of these saponins, reaching levels 10 times higher than those set by the standards of the Chinese Pharmacopoeia (2020 version) due to this species is native distribution as well Agriculture Product of Geographical indications. Importantly, both crude extracts and the six purified saponins are effective in inhibiting cell cycle progression in four human cancer cell lines. All six saponins induce the mitochondrial apoptotic pathway, supporting their potent anticancer activities.

Consequently, the rhizomes of *P. polyphylla* var. *yunnanensis* show promising anticancer effects *in vitro* on three liver human cancer cell lines (SMMC-7721, HepG2, and SK-HEP-1) and one NSCLC cell line (A549). The rhizome of cultivated *P. polyphylla* var. *yunnanensis* may become an important plant source for anticancer treatment, which will be benefit to conserve this medicinal plant and heritage related indigenous and local medicine knowledge as well as sustainable development. Moreover, this study underscores how the use of indigenous and local medicine knowledge of *P. polyphylla* var. *yunnanensis*. Moreover, as a case study, this study provides a new perspective from an ethnobotanical approach to explore anticancer medicine as well as heritage rapidly disappearing indigenous medicine knowledge.

## Data Availability

The original contributions presented in the study are included in the article/Supplementary Material, further inquiries can be directed to the corresponding authors.

## Abbreviations

^13^C-NMR, ^13^C nuclear magnetic resonance; ^1^H-NMR, ^1^H nuclear magnetic resonance; CCCP, carbonyl cyanide 3-chlorophenylhydrazone; DMSO, dimethyl sulfoxide; DOX, doxorubicin; ESI-MS, electrospray ionization–mass spectrometry; FITC, fluorescein isothiocyanate; GAP, good agriculture practice; HCC, hepatocellular carcinoma; HPLC, high-performance liquid chromatography; IC_50_, half maximal inhibitory concentration; JC-10, 5,6-dichloro-1,1′,3,3′-tetraethyl-imidacarbocyanine iodide; MS, mass spectrometry; MTS, 3-(4,5-dimethylthiazol-2-yl)-5(3-carboxymethoxyphenyl)-2-(4-sulfopheny)-2H-tetrazolium; NC, negative control; NMR, nuclear magnetic resonance; NSCLC, non-small-cell lung cancer; PBS, phosphate-buffered saline; PI, propidium iodide; TCM, traditional Chinese medicine; TLC, thin-layer chromatography; TMS, tetramethylsilane; UV, ultraviolet.
