# Supplementary material for: Cytotoxic and pro-apoptotic effects of botanical drugs derived from the indigenous cultivated medicinal plant Paris polyphylla var. yunnanensis
Source: Front Pharmacol. 2023 Jan 26;14:1100825. doi: 10.3389/fphar.2023.1100825 (PMC9911168; doi:10.3389/fphar.2023.1100825)
Supplement: Supplementary file 1 [file DataSheet2.PDF]

1. Flow chart of extraction and isolation in *Paris polyphylla* var. *yunnanensis*.

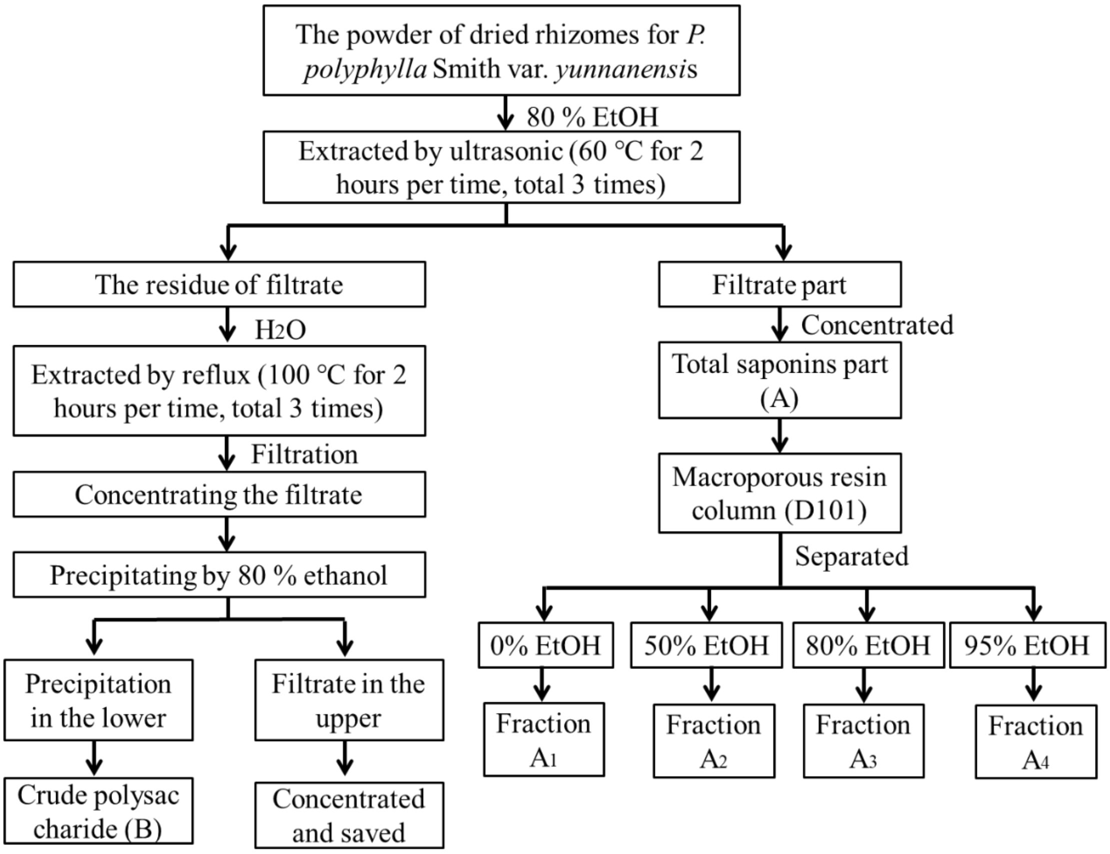

Figure S7 Flow chart of extraction in *Paris polyphylla* var. *yunnanensis*.

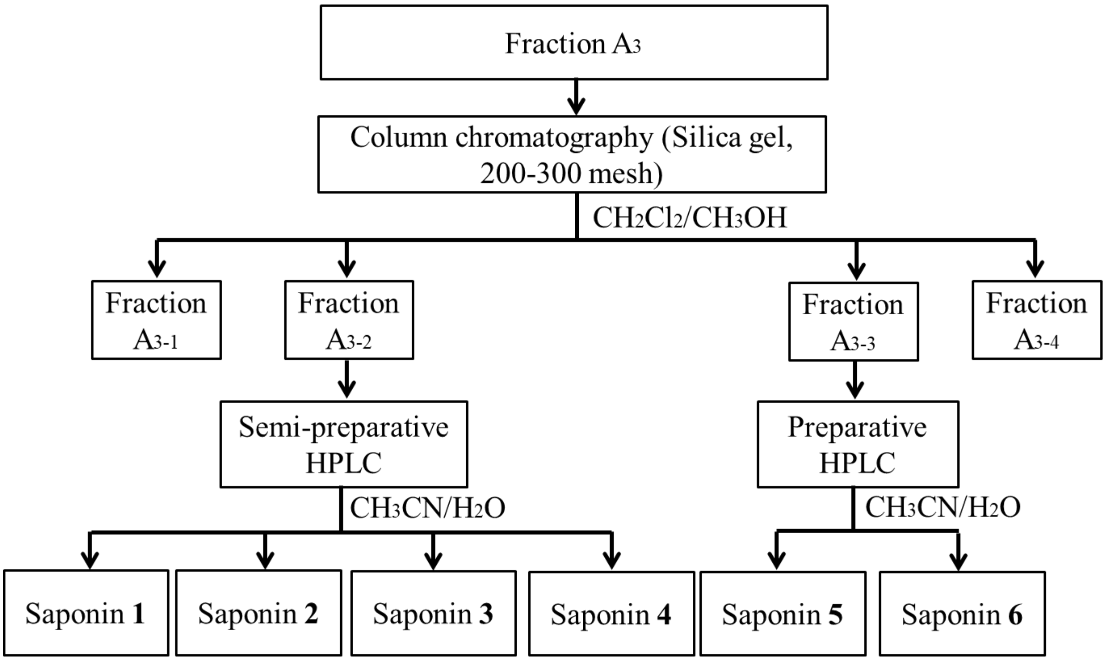

Figure S8 Flow chart of isolation for six saponins.

2. The analysis of mitochondrial membrane potential.

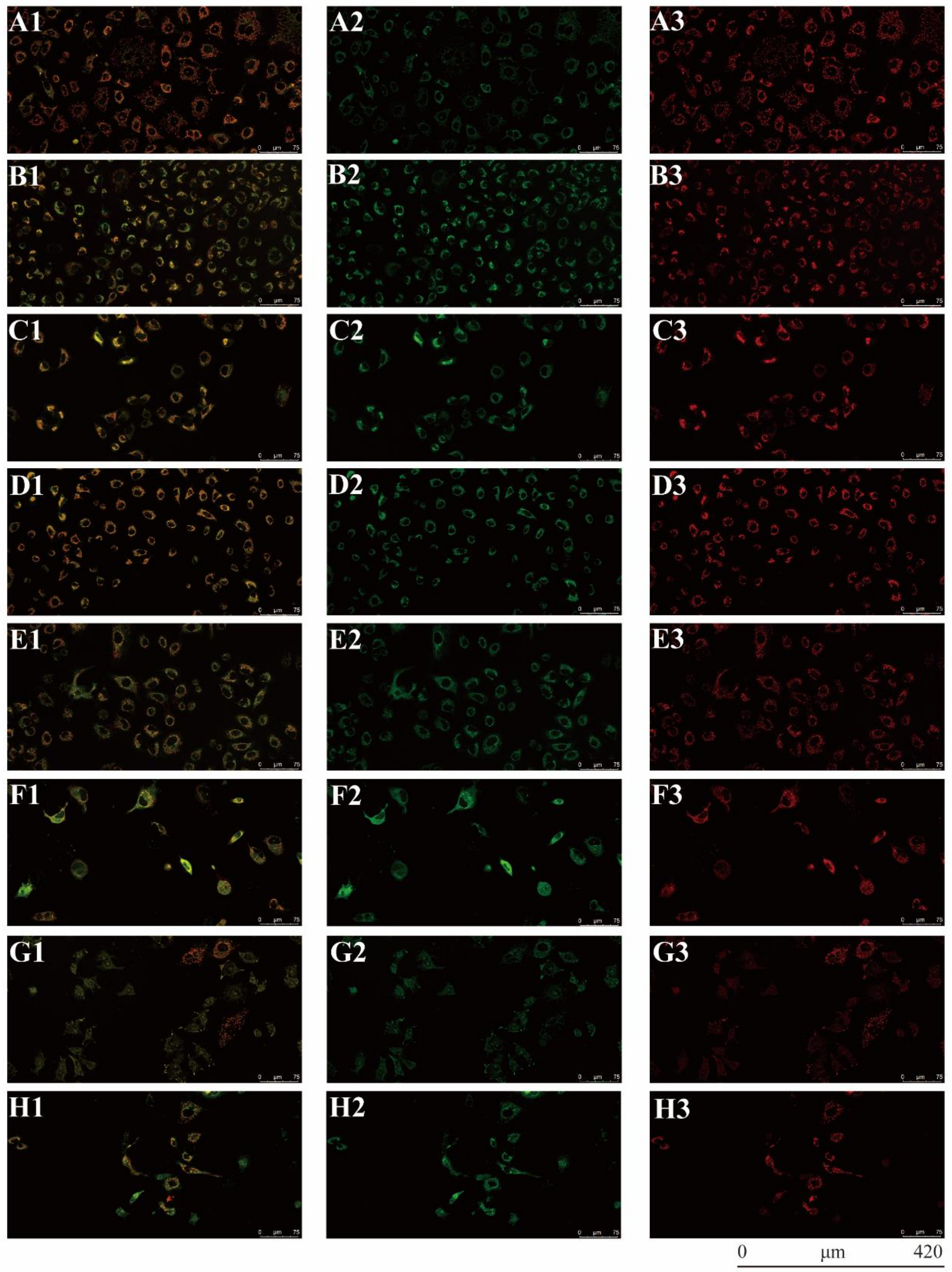

Figure S9 Six saponins affect the mitochondrial membrane potential of A549 cells. **A1** to **H1**, merge by red and green fluorescence. **A2** to **H2**, green fluorescence. **A3** to **H3**, red fluorescence. Untreated groups served as negative control (A1–A3). CCCP-treated groups served as a positive control at 2 μM (B1–B3). All saponins were added at a concentration of 2 μM: saponin **1** (C1–C3), saponin **2** (D1–D3), saponin **3** (E1–E3), saponin **4** (F1–F3), saponin **5** (G1–G3), and saponin **6** (H1–H3).

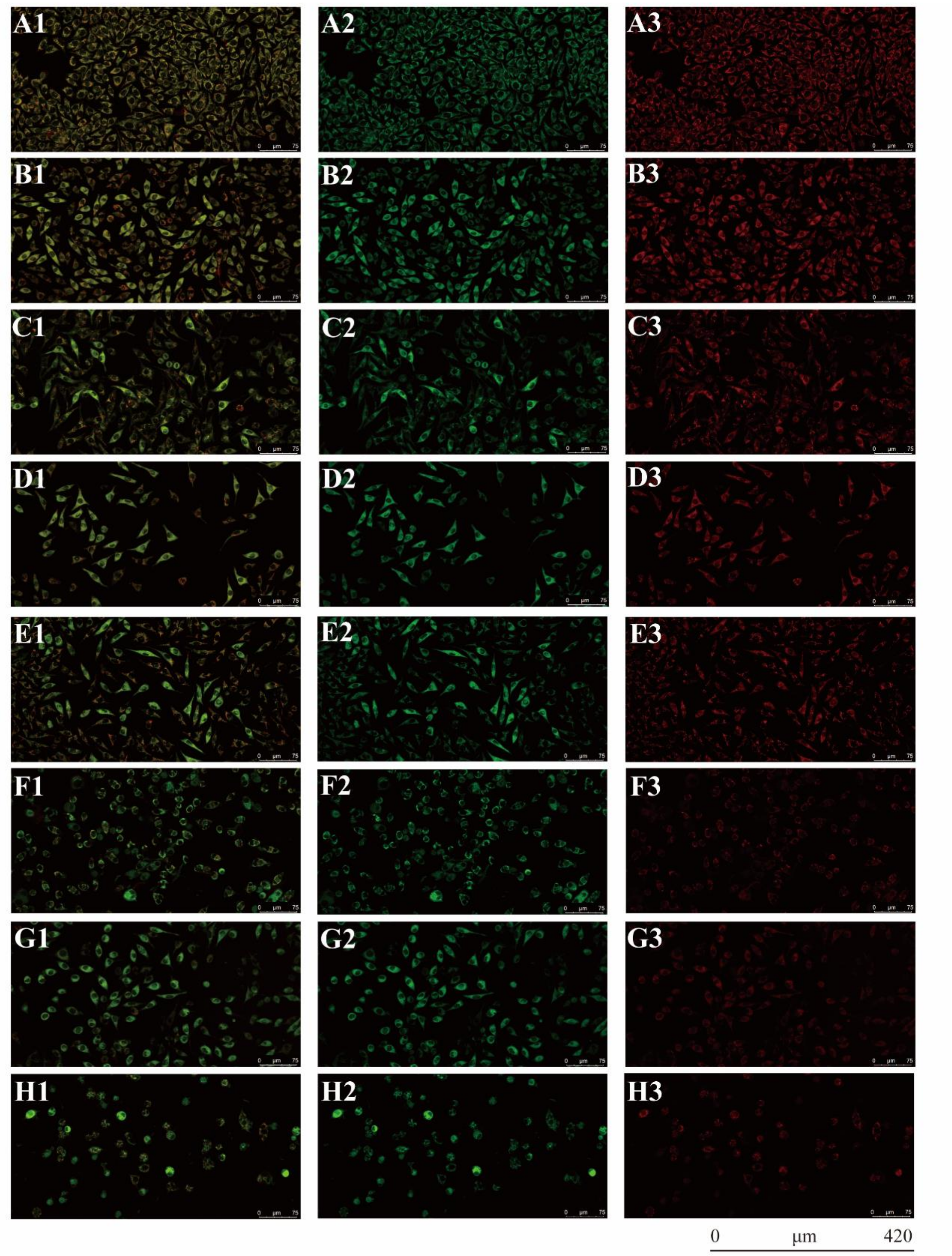

Figure S10 Six saponins affect the mitochondrial membrane potential of SMMC-7721 cells.

A1 to **H1**, merge by red and green fluorescence. **A2** to **H2**, green fluorescence. **A3** to **H3**, red fluorescence. Untreated groups served as negative control (A1–A3). CCCP-treated groups served as a positive control at 2 μM (B1–B3). All saponins were added at a concentration of 2 μM: saponin **1** (C1–C3), saponin **2** (D1–D3), saponin **3** (E1–E3), saponin **4** (F1–F3), saponin **5** (G1–G3), and saponin **6** (H1–H3).

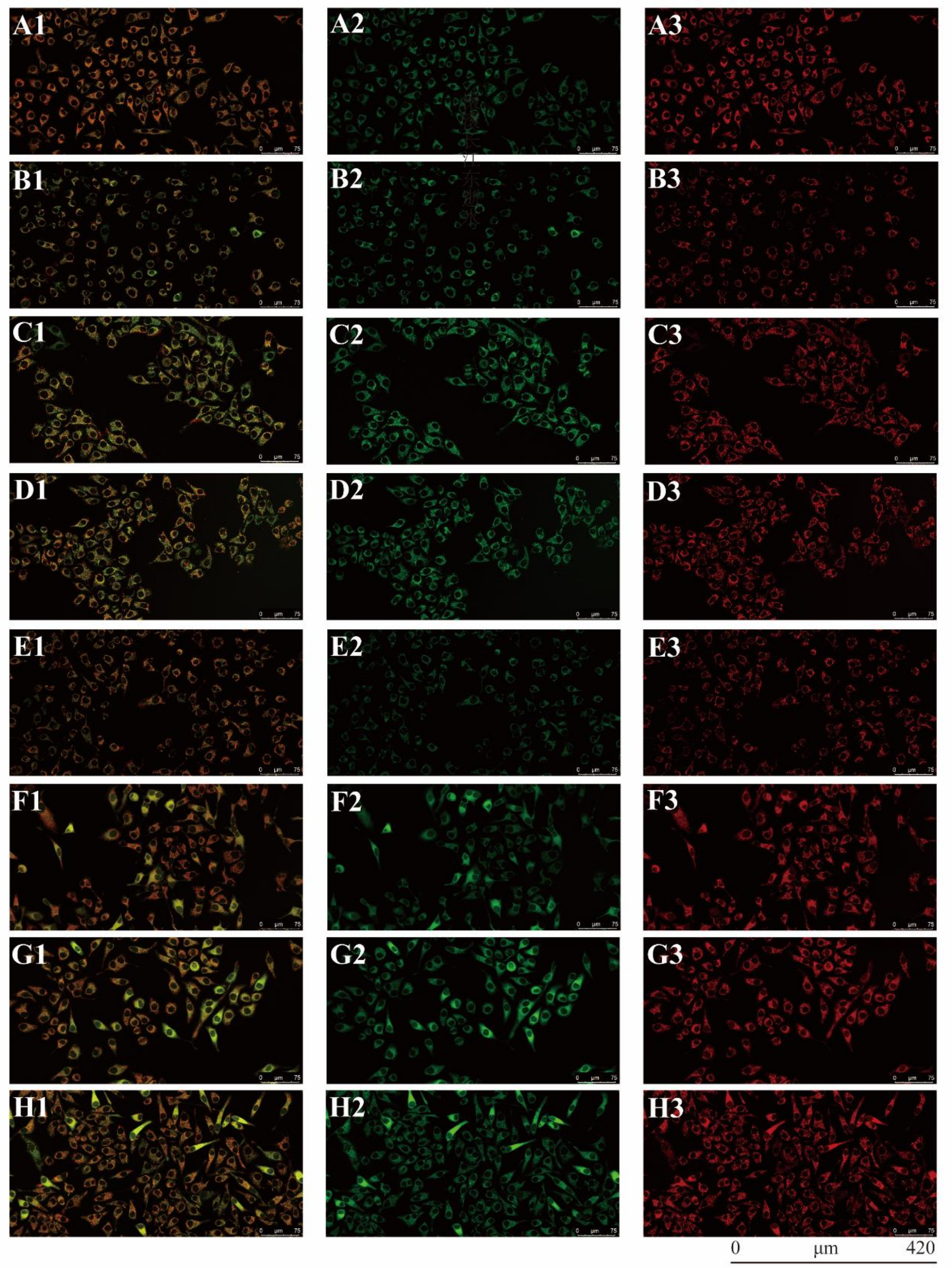

Figure S11 Six saponins affect the mitochondrial membrane potential of HepG2 cells. **A1** to **H1**, merge by red and green fluorescence. **A2** to **H2**, green fluorescence. **A3** to **H3**, red fluorescence. Untreated groups served as negative control (A1–A3). CCCP-treated groups served as a positive control at 2 μM (B1–B3). All saponins were added at a concentration of 2 μM: saponin **1** (C1–C3), saponin **2** (D1–D3), saponin **3** (E1–E3), saponin **4** (F1–F3), saponin **5** (G1–G3), and saponin **6** (H1–H3).
