# Supplementary figures and images for: Cytotoxic and pro-apoptotic effects of botanical drugs derived from the indigenous cultivated medicinal plant Paris polyphylla var. yunnanensis
Source: Front Pharmacol. 2023 Jan 26;14:1100825. doi: 10.3389/fphar.2023.1100825 (PMC9911168; doi:10.3389/fphar.2023.1100825)

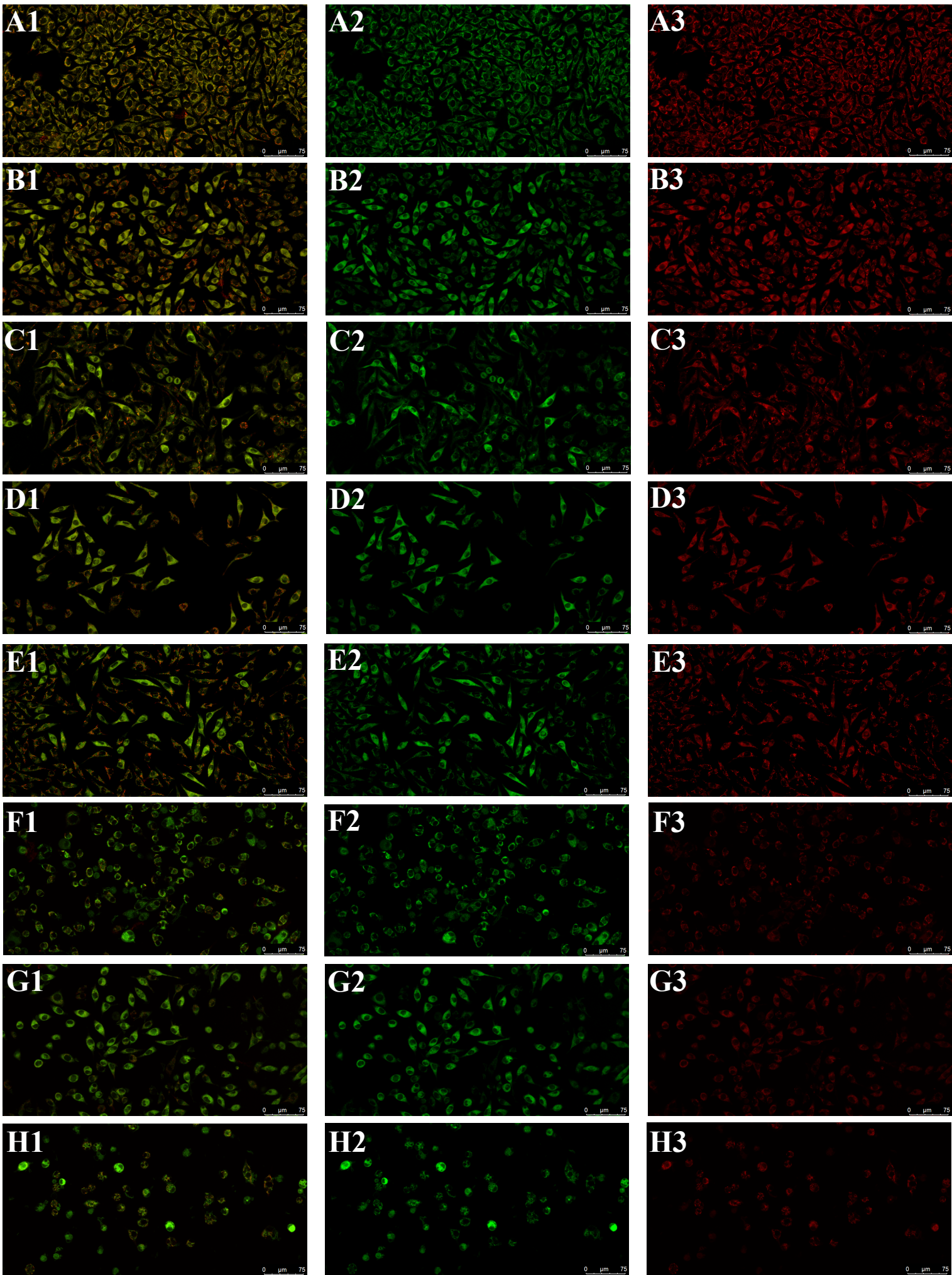

Supplement: Supplementary file 2 [file DataSheet4.PDF]

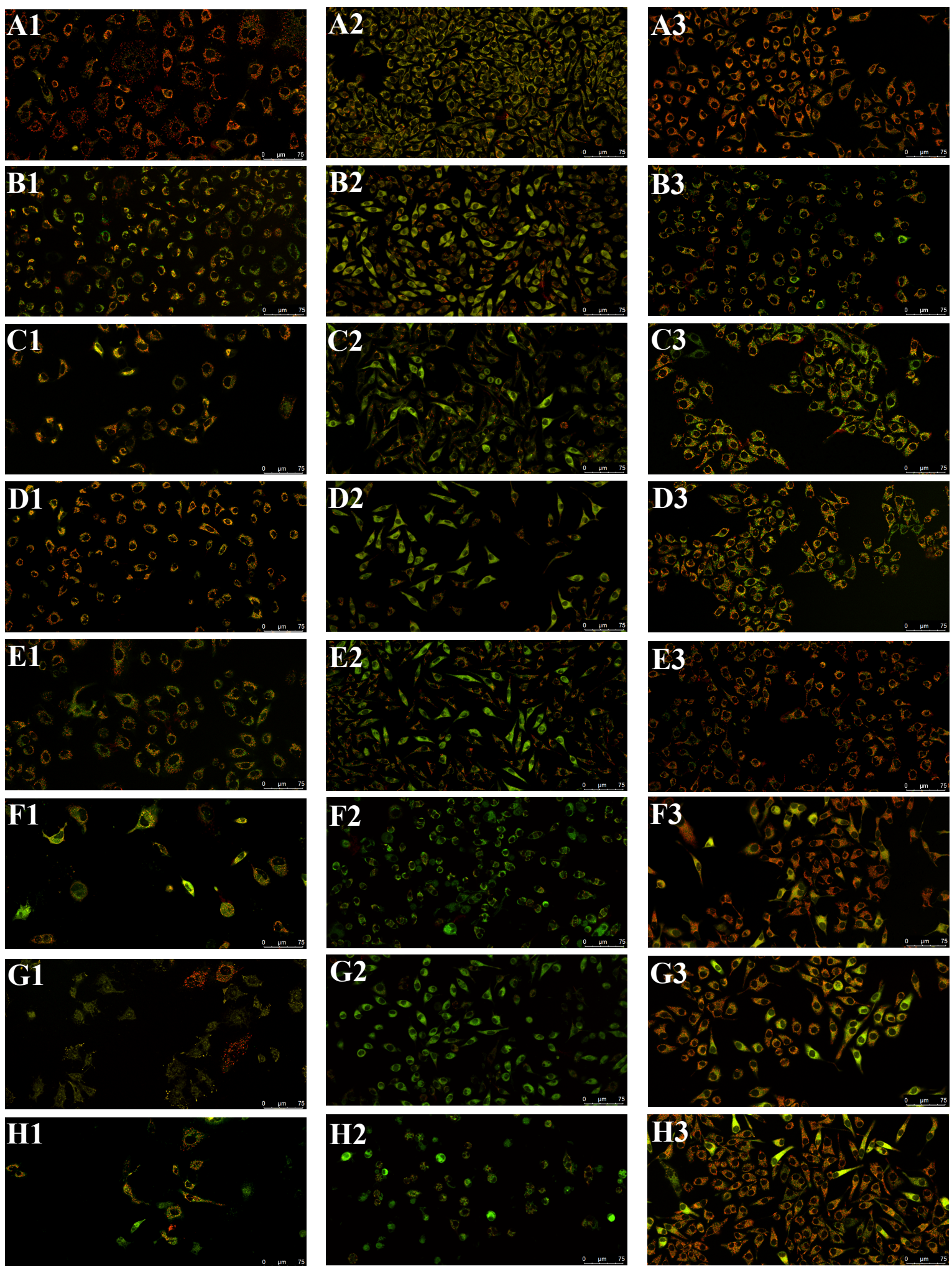

0 μm 420

Supplement: Supplementary file 3 [file DataSheet6.PDF]

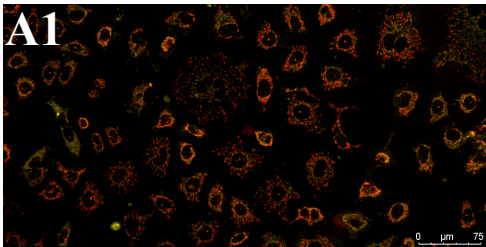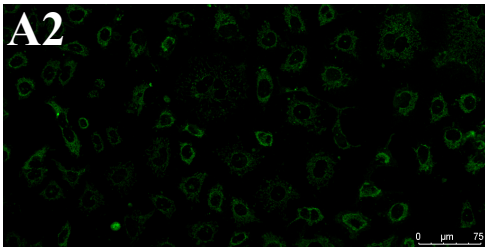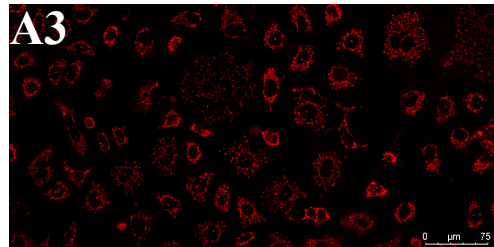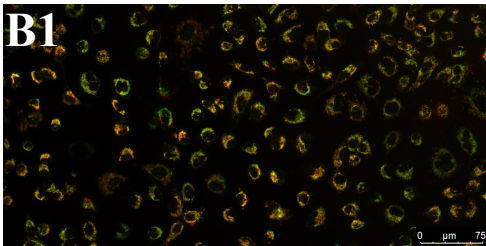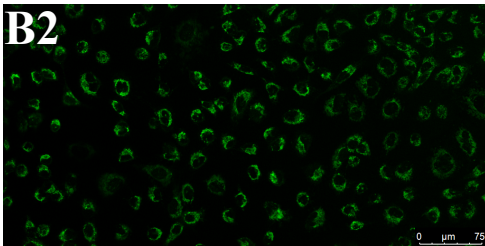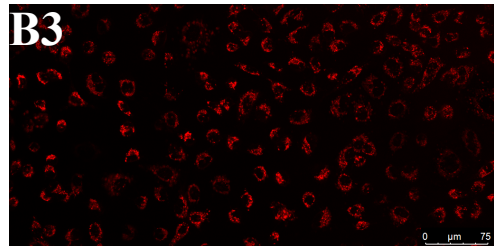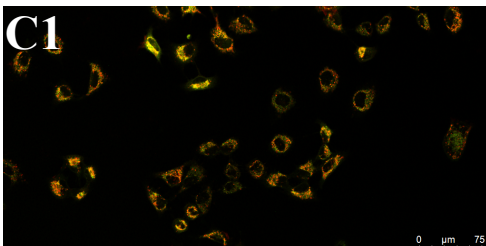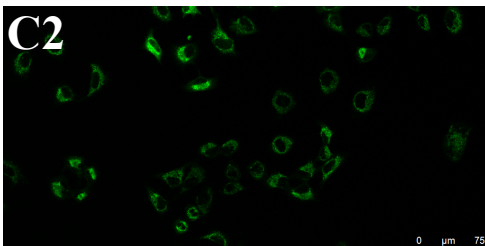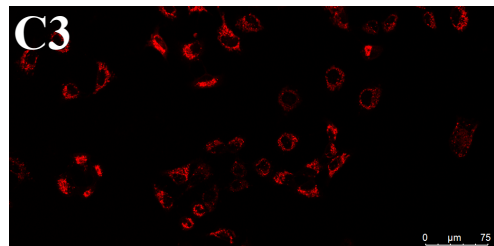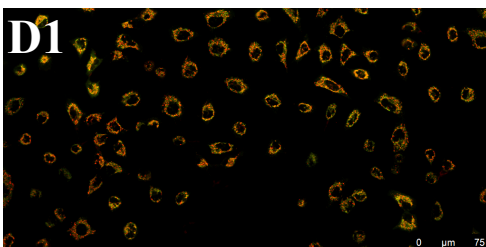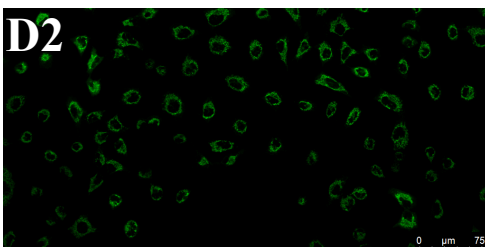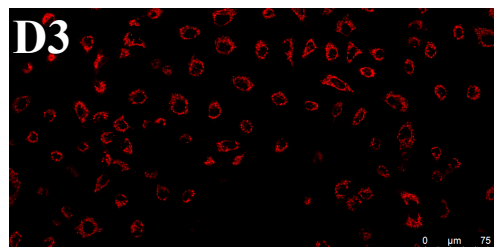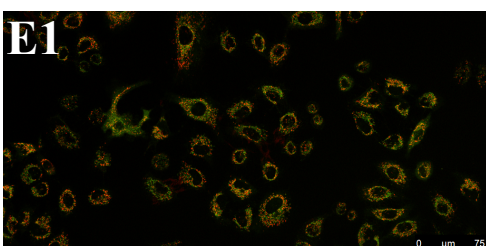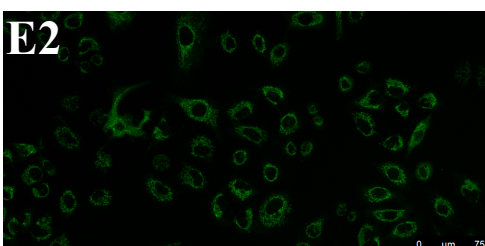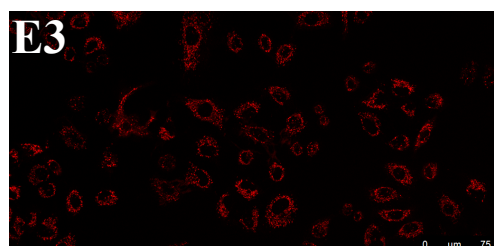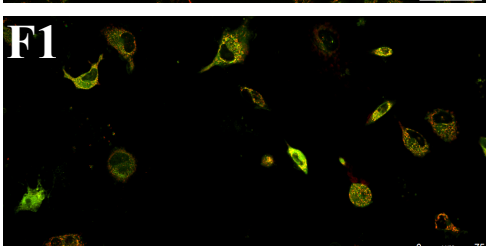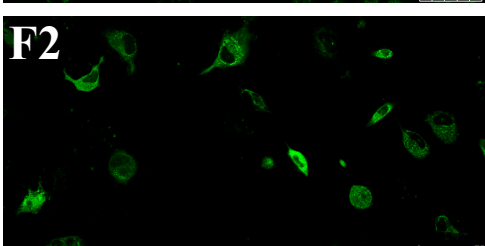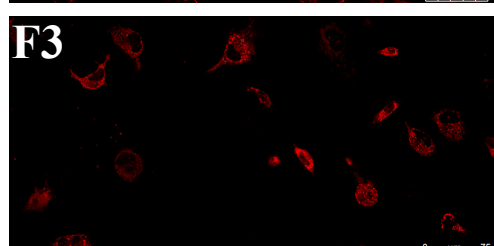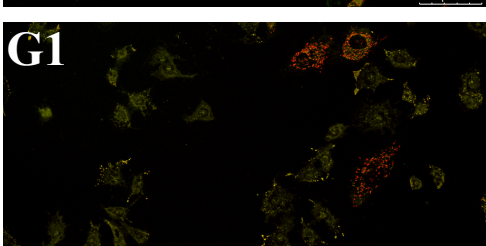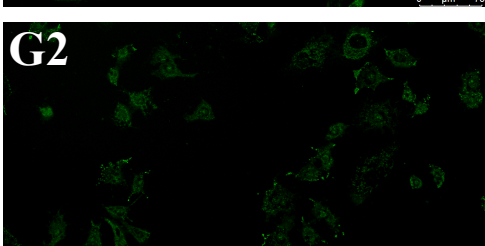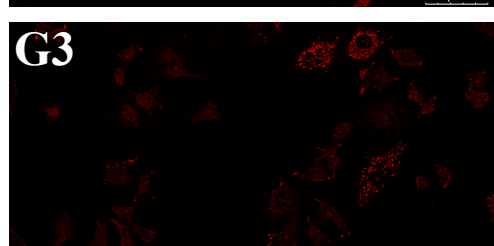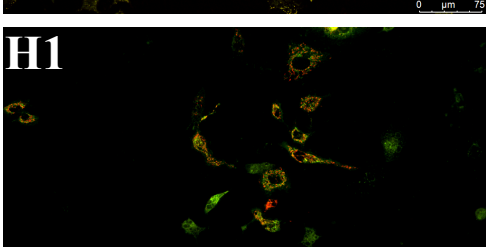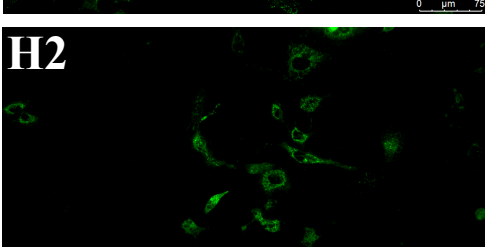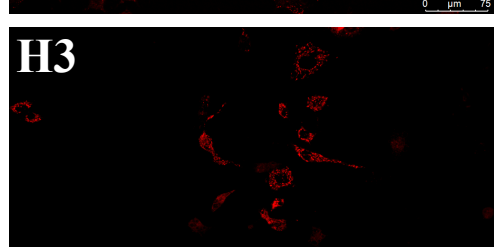

Supplement: Supplementary file 4 [file DataSheet3.PDF]

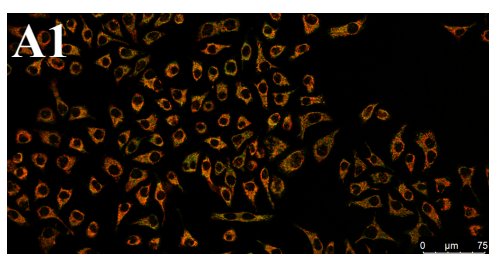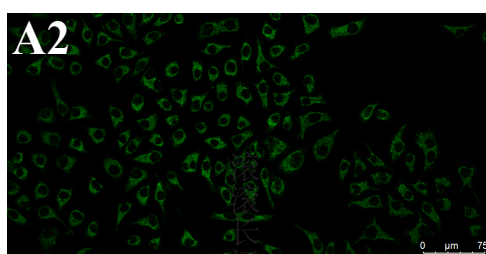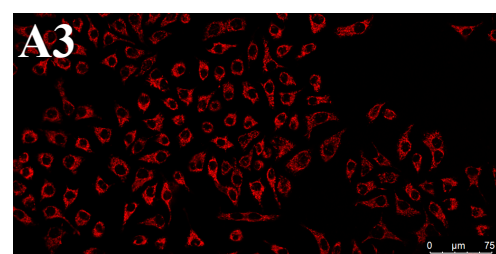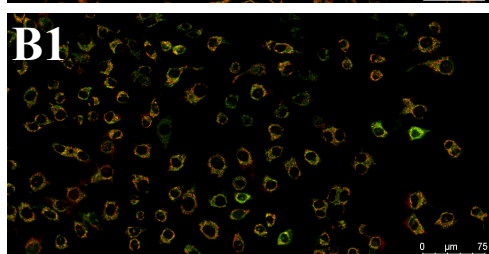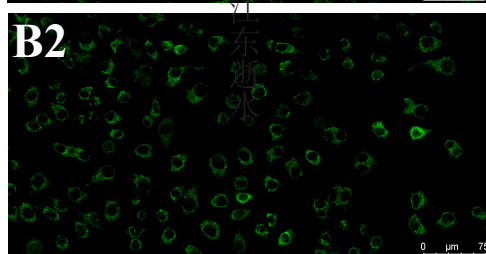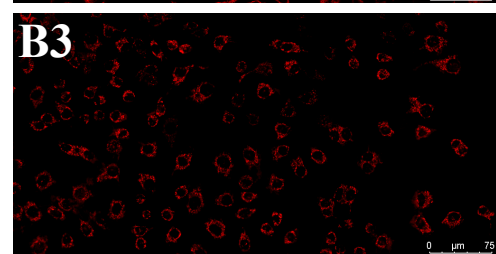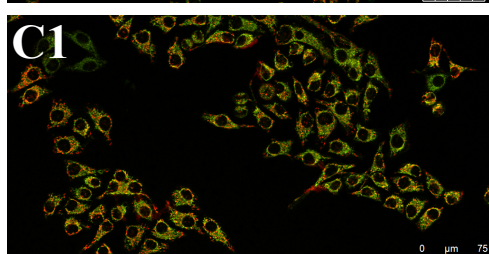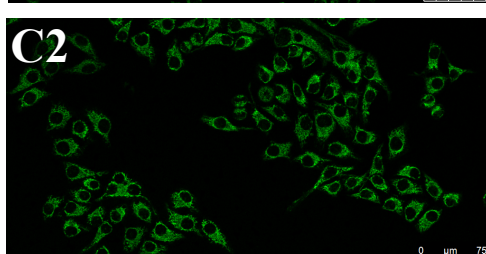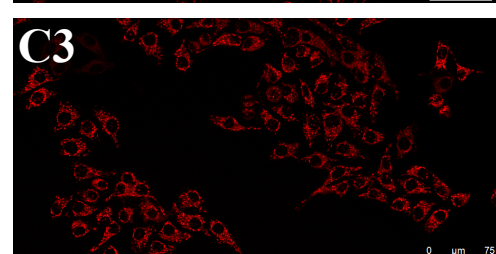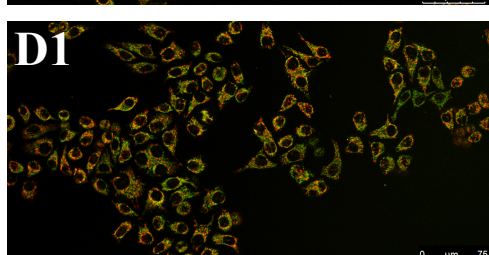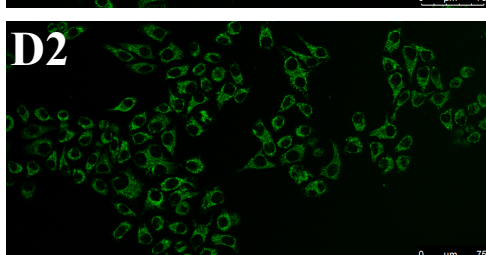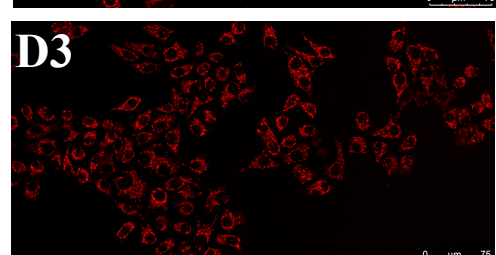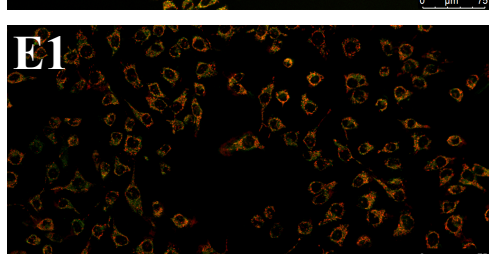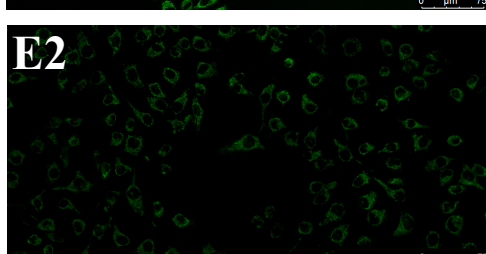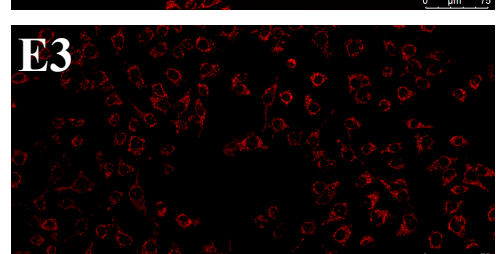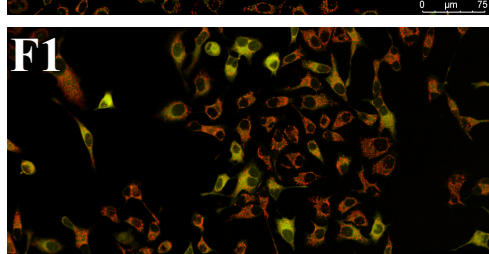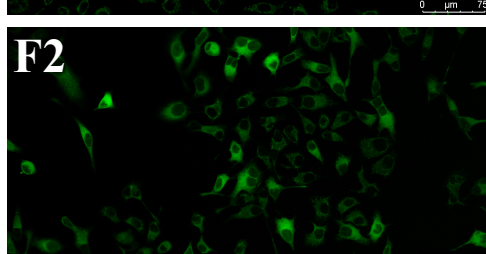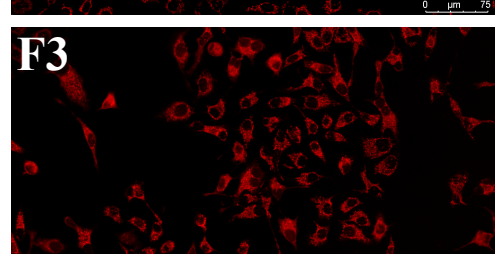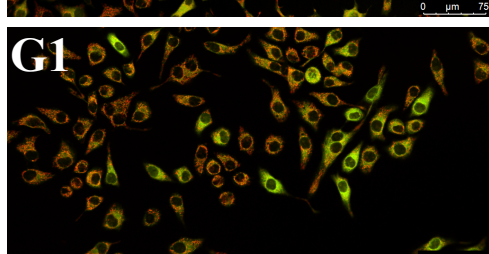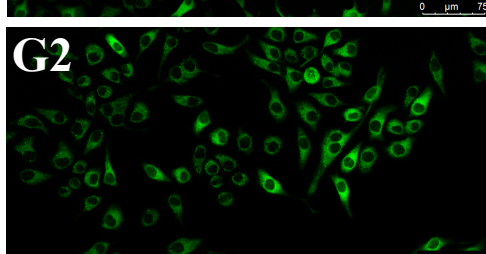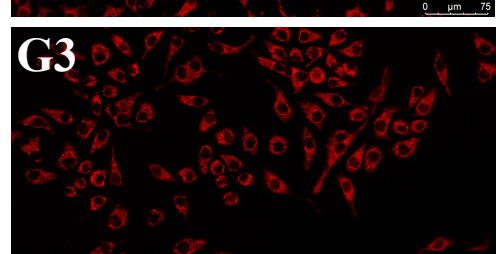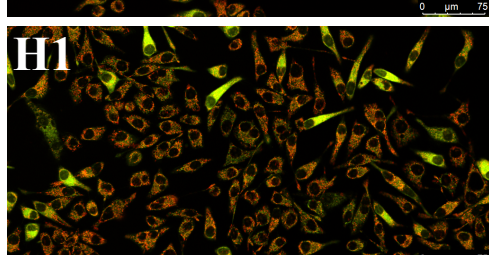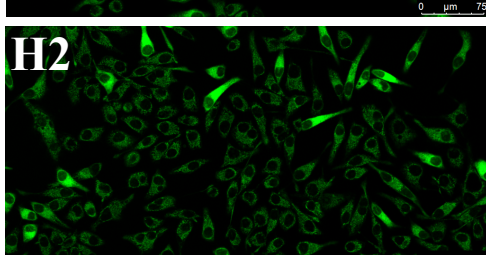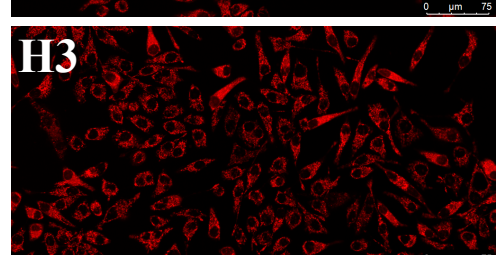0  $\mu\text{m}$  420

Supplement: Supplementary file 6 [file DataSheet5.PDF]
