# Supplementary material for: Cytotoxic and pro-apoptotic effects of botanical drugs derived from the indigenous cultivated medicinal plant Paris polyphylla var. yunnanensis
Source: Front Pharmacol. 2023 Jan 26;14:1100825. doi: 10.3389/fphar.2023.1100825 (PMC9911168; doi:10.3389/fphar.2023.1100825)
Supplement: Supplementary file 5 [file DataSheet1.PDF]

1. Saponin 1

Saponin **1**: White solid, $C_{45}H_{72}O_{17}$; CAS No., 55916-52-4; Paris saponin Pb;
R_f = 0.3 ($CH_2Cl_2/CH_3OH/CH_3COOH$ = 8/2/0.1)
^{1}H-NMR (600 MHz, C_5D_5N) δ: 0.45 (3H, d, CH_3-27), 0.72 (3H, s, CH_3-18), 0.84 (3H, s, CH_3-19), 1.00 (3H, d, CH_3-21), 1.39 (3H, d, CH_3-Rha II), 1.53 (3H, d, CH_3-Rha I), 4.61 (1H, d, Glc-H-1), 5.07 (1H, br, H-6), 5.62 (1H, s, Rha II-H-1), 6.16 (1H, s, Rha I-H-1), (See Figure S1-1).
^{13}C-NMR (600 MHz, C_5D_5N) δ: 141.9 (C-5), 122.6 (C-6), 110.6 (C-22), 103.6 (Rha II-C1), 102.8 (Rha I-C1), 101.0 (Glc-C1), 90.9 (C-16), 90.7 (C-17), 79.3 (Glc-C4), 78.8 (C-3), 78.7 (Glc-C2), 78.5 (Glc-C3), 77.6 (Glc-C5), 74.8 (Rha I-C4), 74.6 (Rha II-C4), 73.6 (Rha I-C3), 73.5 (Rha II-C3), 73.2 (Rha I-C2), 73.2 (Rha II-C2), 71.1 (Rha II-C5), 70.3 (Rha I-C5), 67.4 (C-26), 62.0 (Glc-C6), 53.8 (C-14), 51.0 (C-9), 45.9 (C-13), 45.5 (C-20), 39.7 (C-4), 38.3 (C-1), 37.9 (C-10), 33.2 (C-7), 33.1 (C-12), 32.8 (C-15), 32.5 (C-23), 31.2 (C-8), 30.9 (C-25), 30.7 (C-2), 29.5 (C-24), 21.7 (C-11), 20.2 (C-19), 19.4 (Rha I-C6), 19.2 (Rha II-C6), 18.0 (C-27), 17.9 (C-18), 10.5 (C-21), (See Figure S1-2).
ESI-MS *m/z* 907 $[M+Na]^+$ (See Figure S1-3).

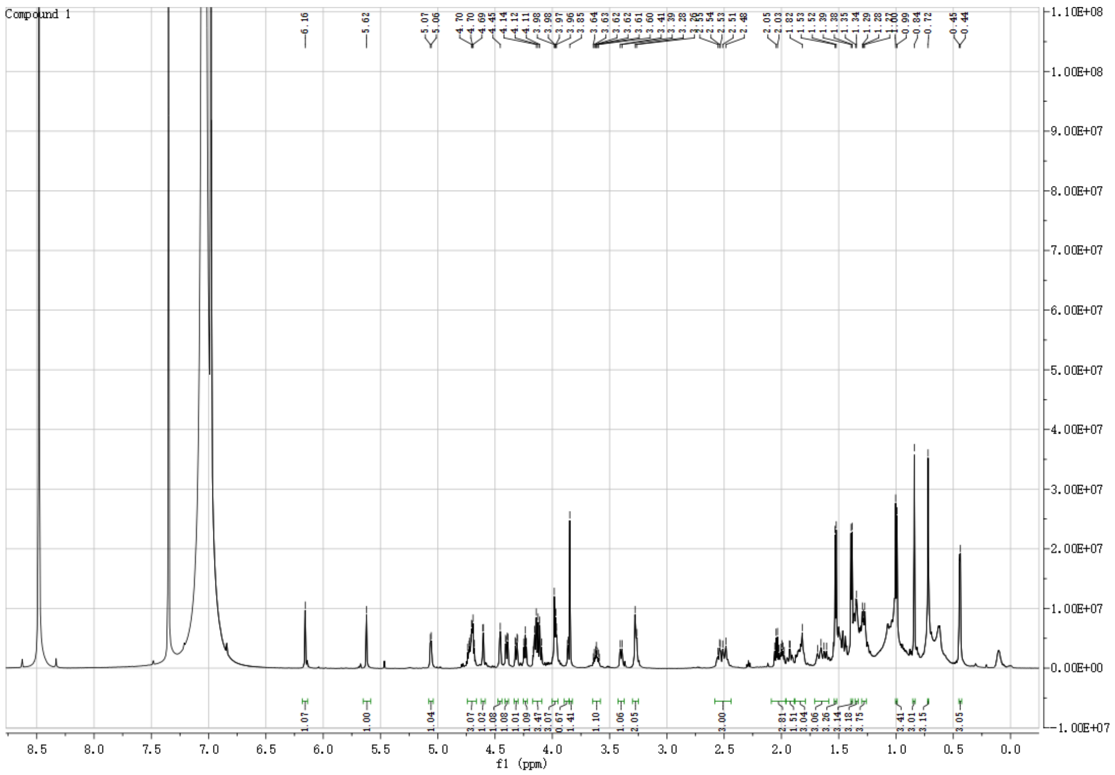

Figure S1-1 ^{1}H NMR Spectra for Saponin **1**.

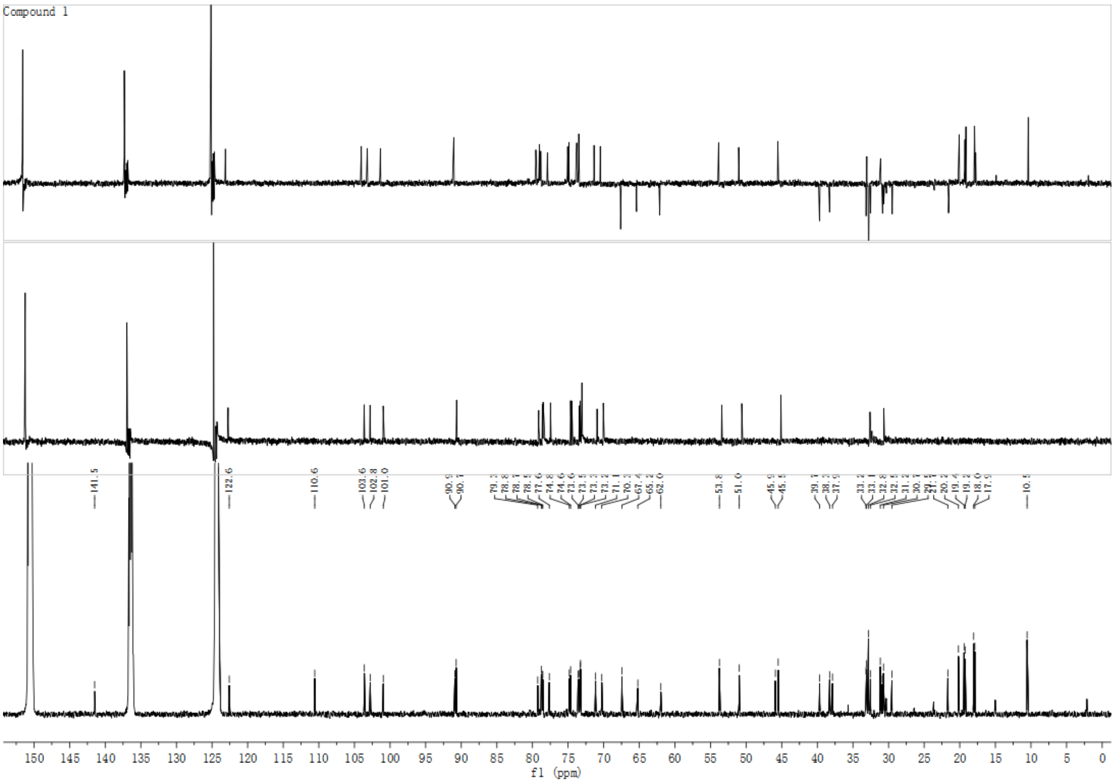

Figure S1-2 ^{13}C NMR Spectra for Saponin **1**.

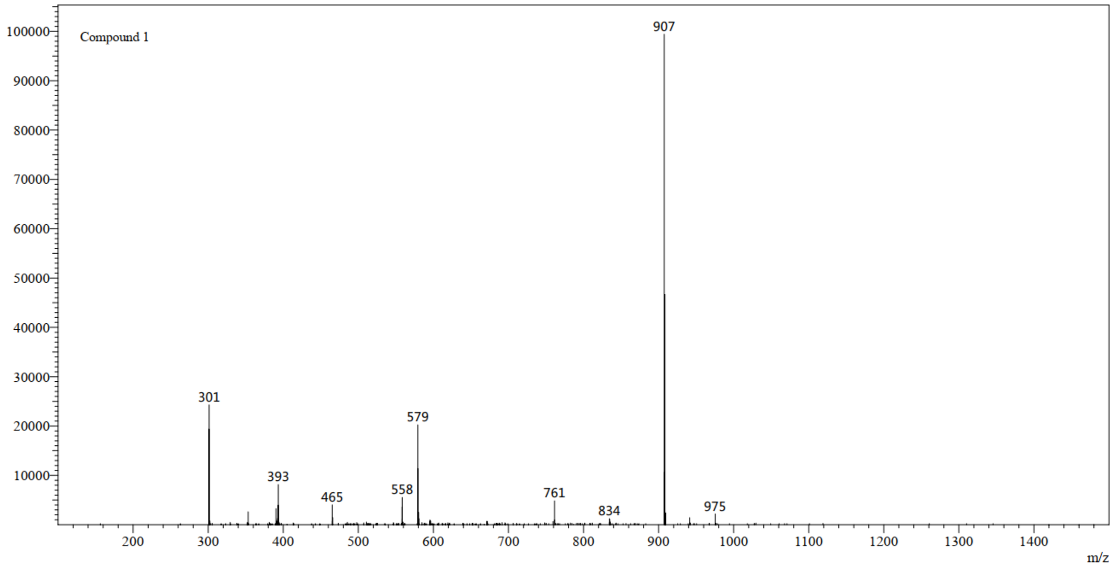

Figure S1-3 ESI-MS Spectra for Saponin **1**.

2. Saponin 2

Saponin **2**: White solid, $C_{44}H_{70}O_{17}$; CAS No., 81917-50-2; Paris saponin H;
R_f = 0.3 ($CH_2Cl_2/CH_3OH/CH_3COOH$ = 8/2/0.1)
^{1}H-NMR (600 MHz, C_5D_5N) δ: 0.47 (3H, d, CH_3-27), 0.74 (3H, s, CH_3-18), 0.86 (3H, s, CH_3-19), 1.01 (3H, d, CH_3-21), 1.56 (3H, d, CH_3-Rha), 4.57 (1H, d, Glc-H-1), 5.07 (1H, br, H-6), 5.71 (1H, s, Ara-H-1), 6.06 (1H, s, Rha-H-1), (See Figure S2-1).
^{13}C-NMR (600 MHz, C_5D_5N) δ: 141.5 (C-5), 122.8 (C-6), 110.6 (C-22), 110.4 (Ara-C1), 102.7 (Rha-C1), 100.9 (Glc-C1), 90.9 (C-16), 90.8 (C-17), 87.4 (Ara-C4), 83.5 (Ara-C2), 78.8 (C-3), 78.7 (Ara-C3), 78.5 (Glc-C3), 78.2 (Glc-C2), 77.8 (Glc-C5), 77.4 (Glc-C4), 74.9 (Rha-C4), 73.6 (Rha-C3), 73.2 (Rha-C2), 70.3 (Rha-C5), 67.5 (C-26), 63.3 (Ara-C5), 62.1 (Glc-C6), 53.8 (C-14), 51.0 (C-9), 45.9 (C-13), 45.5 (C-20), 39.7 (C-4), 38.3 (C-1), 37.9 (C-10), 33.2 (C-7), 33.1 (C-12), 32.9 (C-15), 32.6 (C-23), 31.2 (C-8), 30.9 (C-25), 30.7 (C-2), 29.6 (C-24), 21.7 (C-11), 20.2 (C-19), 19.4 (Rha-C6), 18.1 (C-27), 17.9 (C-18), 10.6 (C-21), (See Figure S2-2).
ESI-MS *m/z* 893 $[M+Na]^+$ (See Figure S2-3).

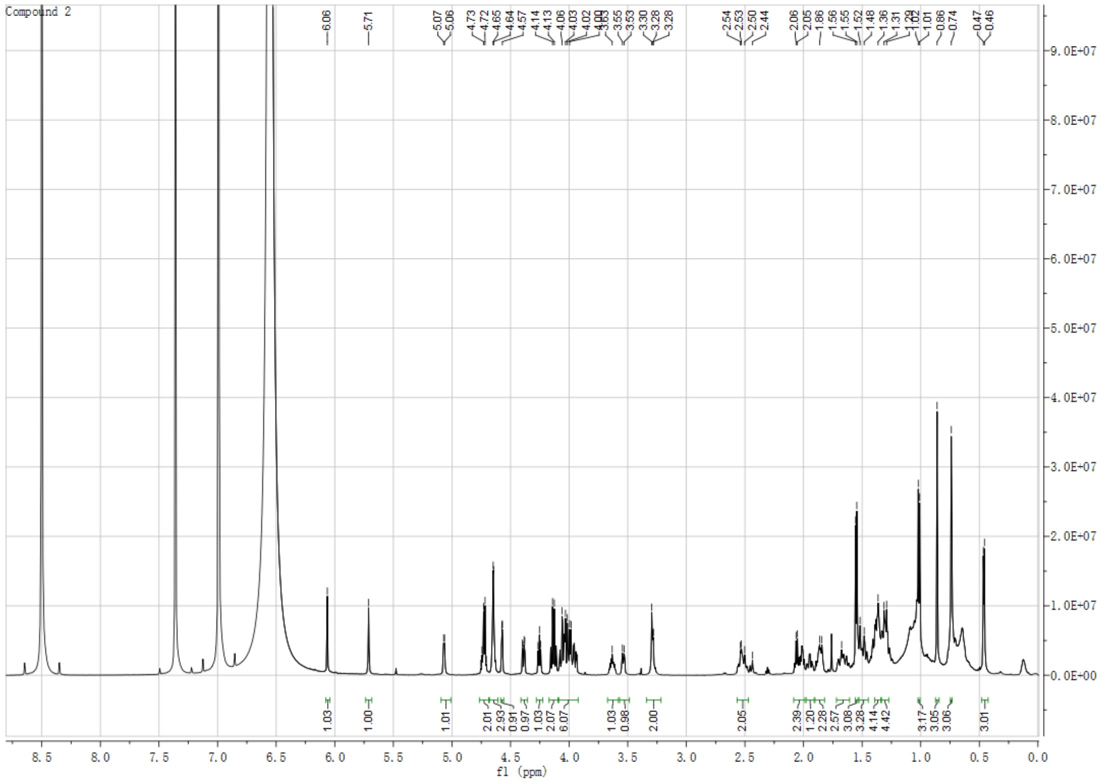

Figure S2-1 ^{1}H NMR Spectra for Saponin **2**.

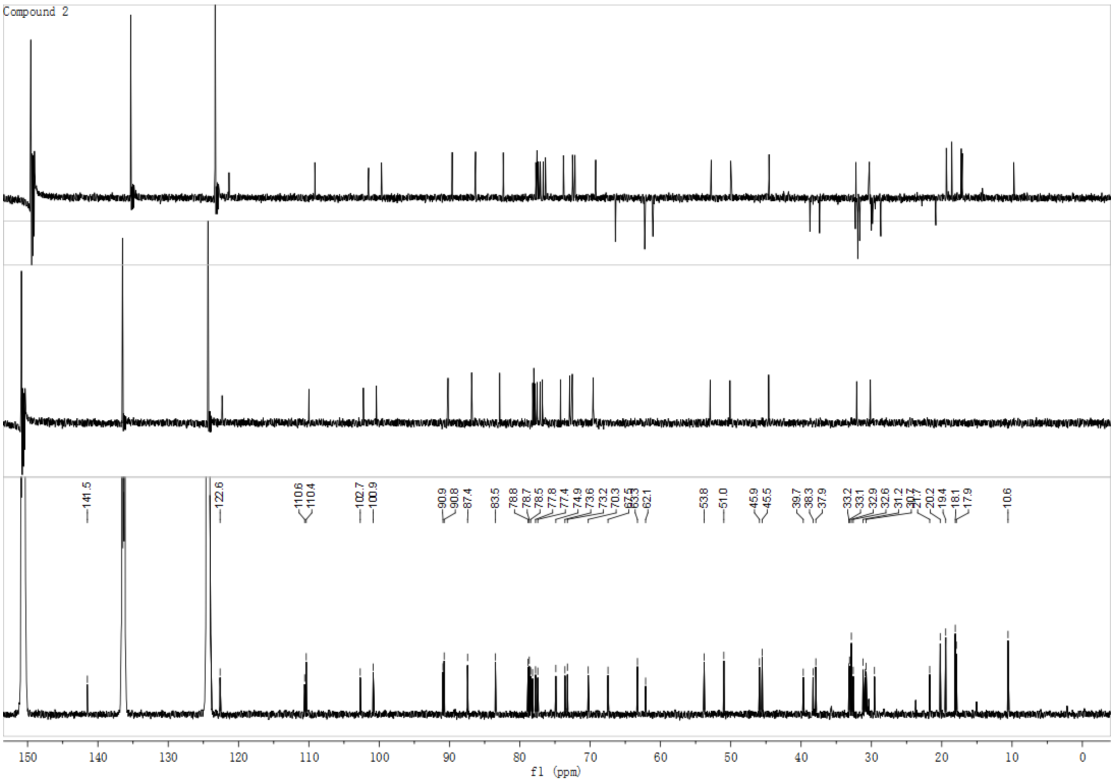

Figure S2-2 ^{13}C NMR Spectra for Saponin **2**.

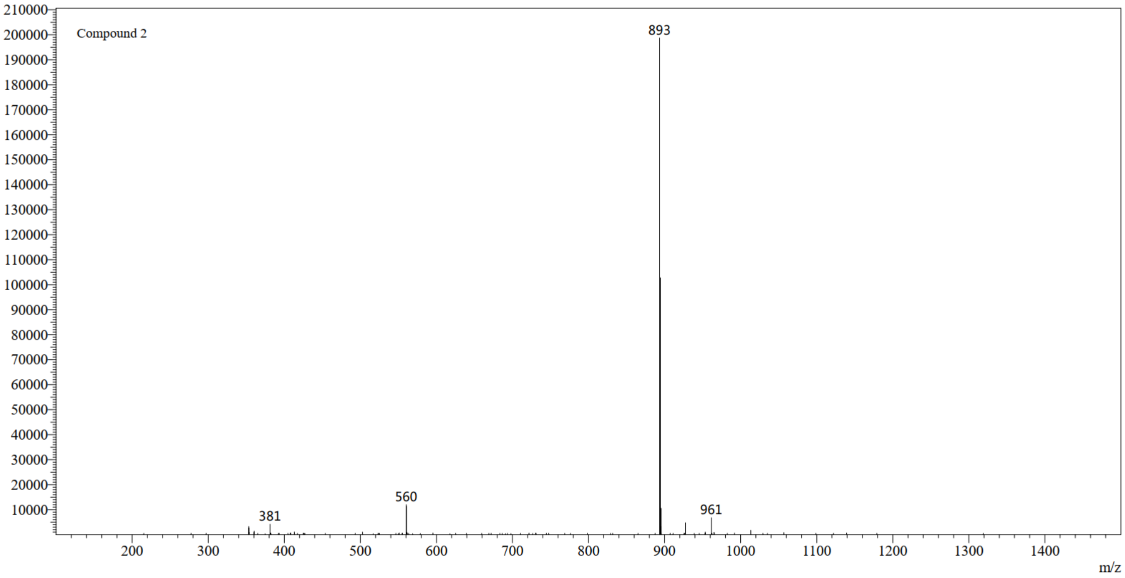

Figure S2-3 ESI-MS Spectra for Saponin **2**.

3. Saponin 3

Saponin **3**: White solid, $C_{45}H_{72}O_{16}$; CAS No., 19057-60-4; Paris saponin III;
R_f = 0.3 ($CH_2Cl_2/CH_3OH/CH_3COOH$ = 8/2/0.1)
^{1}H-NMR (600 MHz, C_5D_5N) δ: 0.48 (3H, d, CH_3-27), 0.61 (3H, s, CH_3-18), 0.83 (3H, s, CH_3-19), 0.92 (3H, d, CH_3-21), 1.42 (3H, d, CH_3-Rha II), 1.56 (3H, d, CH_3-Rha I), 4.63 (1H, d, Glc-H-1), 5.10 (1H, br, H-6), 5.64 (1H, s, Rha II-H-1), 6.18 (1H, s, Rha I-H-1), (See Figure S3-1).
^{13}C-NMR (600 MHz, C_5D_5N) δ: 141.6 (C-5), 122.6 (C-6), 110.1 (C-22), 103.7 (Rha II-C1), 102.8 (Rha I-C1), 101.0 (Glc-C1), 81.9 (C-16), 79.3 (Glc-C4), 78.8 (C-3), 78.7 (Glc-C2), 78.6 (Glc-C3), 77.7 (Glc-C5), 74.9 (Rha I-C4), 74.7 (Rha II-C4), 73.6 (Rha II-C3), 73.5 (Rha I-C3), 73.3 (Rha II-C2), 73.3 (Rha I-C2), 71.2 (Rha II-C5), 70.3 (Rha I-C5), 67.6 (C-26), 63.6 (C-17), 62.0 (Glc-C6), 57.4 (C-14), 51.1 (C-9), 42.7 (C-20), 41.2 (C-13), 40.6 (C-12), 39.7 (C-4), 38.3 (C-1), 37.9 (C-10), 33.1 (C-7), 32.6 (C-15), 32.4 (C-23), 31.4 (C-8), 30.9 (C-25), 30.8 (C-2), 30.0 (C-24), 21.9 (C-11), 20.2 (C-19), 19.4 (Rha I-C6), 19.3 (Rha II-C6), 18.1 (C-27), 17.1 (C-18), 15.8 (C-21), (See Figure S3-2).
ESI-MS *m/z* 891 $[M+Na]^+$ (See Figure S3-3).

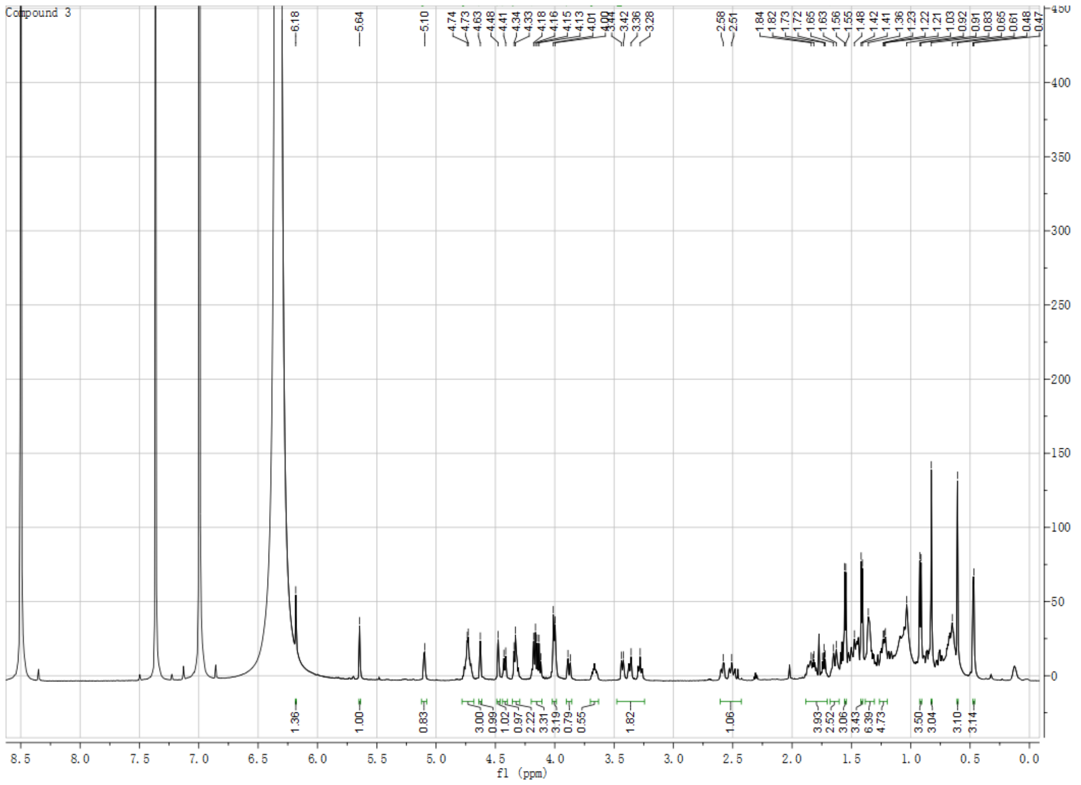

Figure S3-1 ^{1}H NMR Spectra for Saponin **3**.

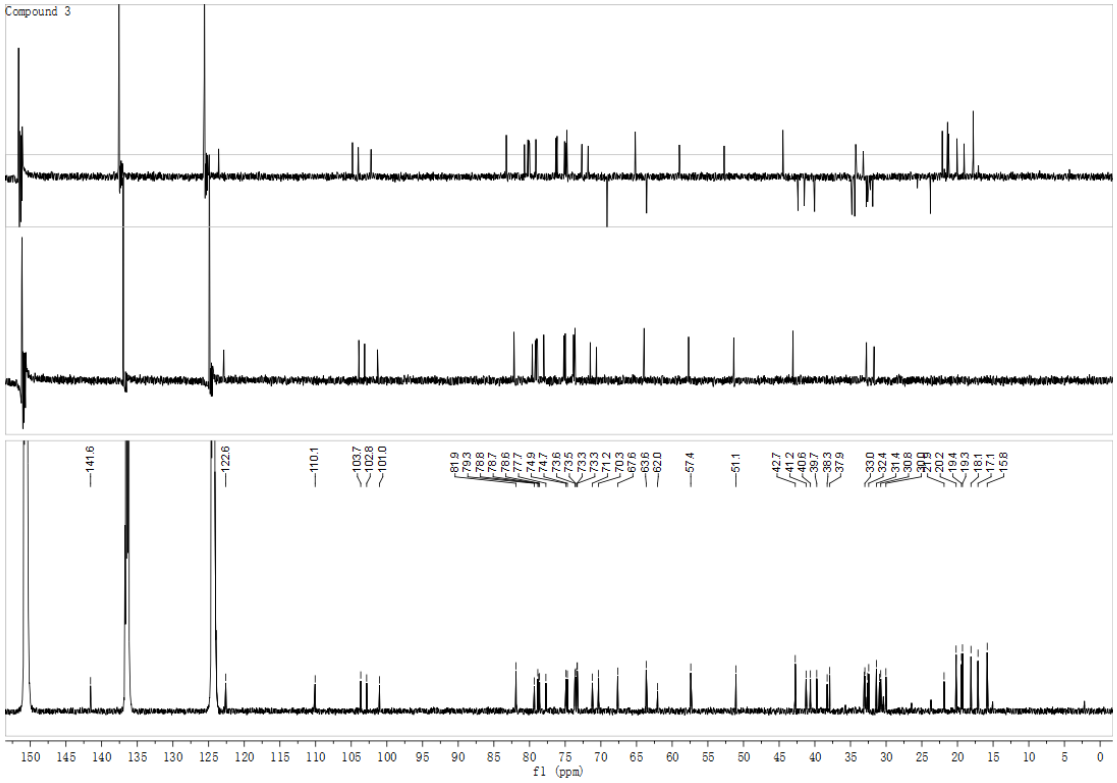

Figure S3-2 ^{13}C NMR Spectra for Saponin **3**.

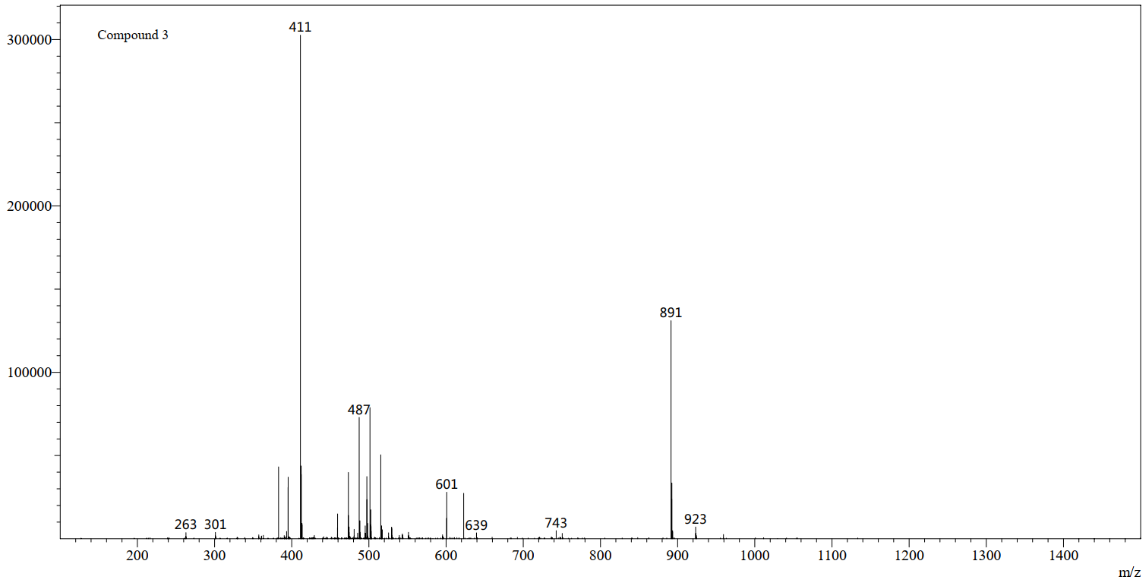

Figure S3-3 ESI-MS Spectra for Saponin **3**.

4. Saponin 4

Saponin **4**: White solid, $C_{44}H_{70}O_{16}$; CAS No., 50773-41-6; Paris saponin I;
R_f = 0.3 ($CH_2Cl_2/CH_3OH/CH_3COOH$ = 8/2/0.1)
^{1}H-NMR (600 MHz, C_5D_5N) δ: 0.48 (3H, d, CH_3-27), 0.61 (3H, s, CH_3-18), 0.83 (3H, s, CH_3-19), 0.92 (3H, d, CH_3-21), 1.56 (3H, d, CH_3-Rha), 4.65 (1H, d, Glc-H-1), 5.10 (1H, br, H-6), 5.71 (1H, s, Ara-H-1), 6.06 (1H, s, Rha-H-1), (See Figure S4-1).
^{13}C-NMR (600 MHz, C_5D_5N) δ: 141.5 (C-5), 122.6 (C-6), 110.4 (Ara-C1), 110.1 (C-22), 102.7 (Rha-C1), 100.9 (Glc-C1), 87.4 (Ara-C4), 83.5 (Ara-C2), 81.9 (C-16), 78.9 (C-3), 78.7 (Ara-C3), 78.5 (Glc-C2), 78.2 (Glc-C3), 77.8 (Glc-C5), 77.5 (Glc-C4), 74.9 (Rha-C4), 73.6 (Rha-C3), 73.2 (Rha-C2), 70.2 (Rha-C5), 67.6 (C-26), 63.6 (Ara-C5), 63.3 (C-17), 62.1 (Glc-C6), 57.4 (C-14), 51.0 (C-9), 42.7 (C-20), 41.2 (C-13), 40.6 (C-12), 39.7 (C-4), 38.3 (C-1), 37.9 (C-10), 33.1 (C-7), 32.6 (C-15), 32.4 (C-23), 31.4 (C-8), 30.9 (C-25), 30.8 (C-2), 30.0 (C-24), 21.9 (C-11), 20.2 (C-19), 19.4 (Rha-C6), 18.1 (C-27), 17.1 (C-18), 15.8 (C-21), (See Figure S4-2).
ESI-MS *m/z* 877 $[M+Na]^+$ (See Figure S4-3).

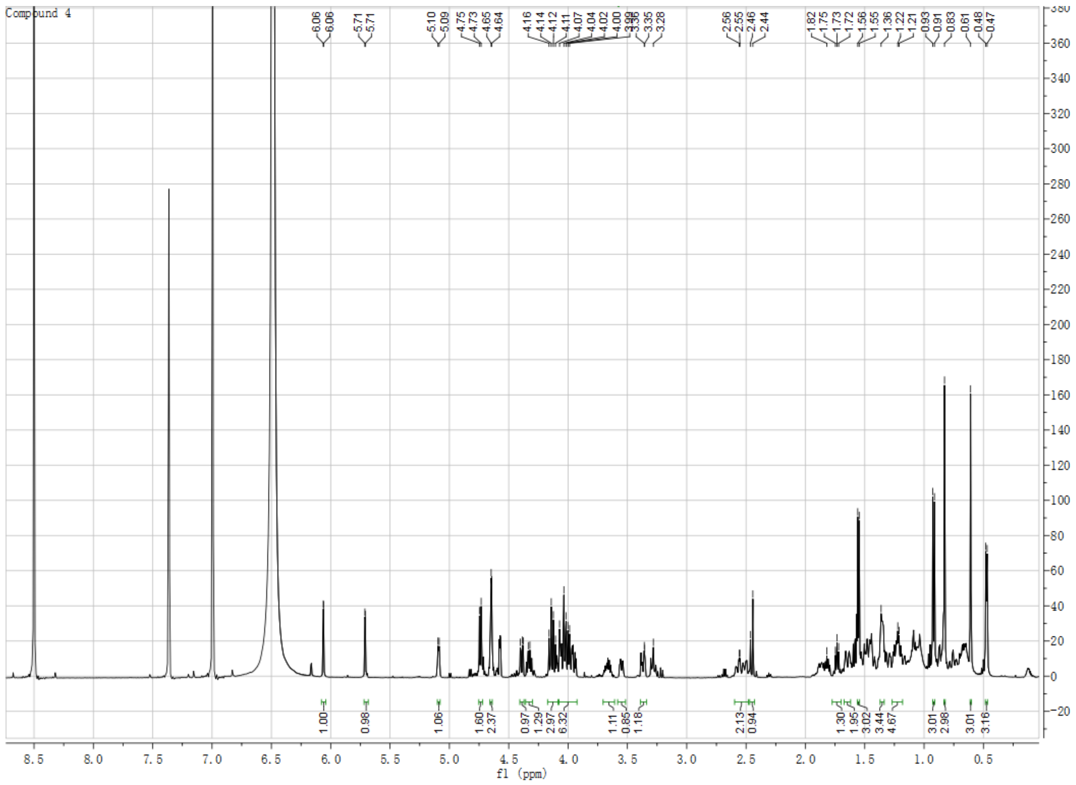

Figure S4-1 ^{1}H NMR Spectra for Saponin **4**.

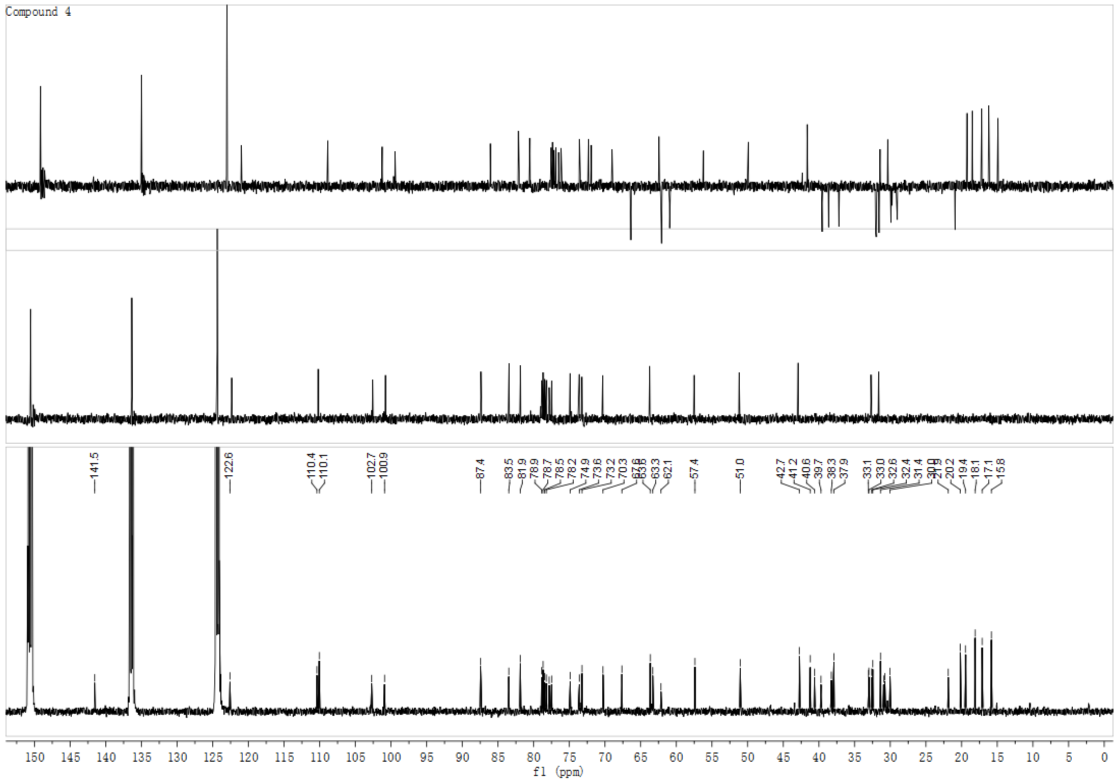

Figure S4-2 ^{13}C NMR Spectra for Saponin **4**.

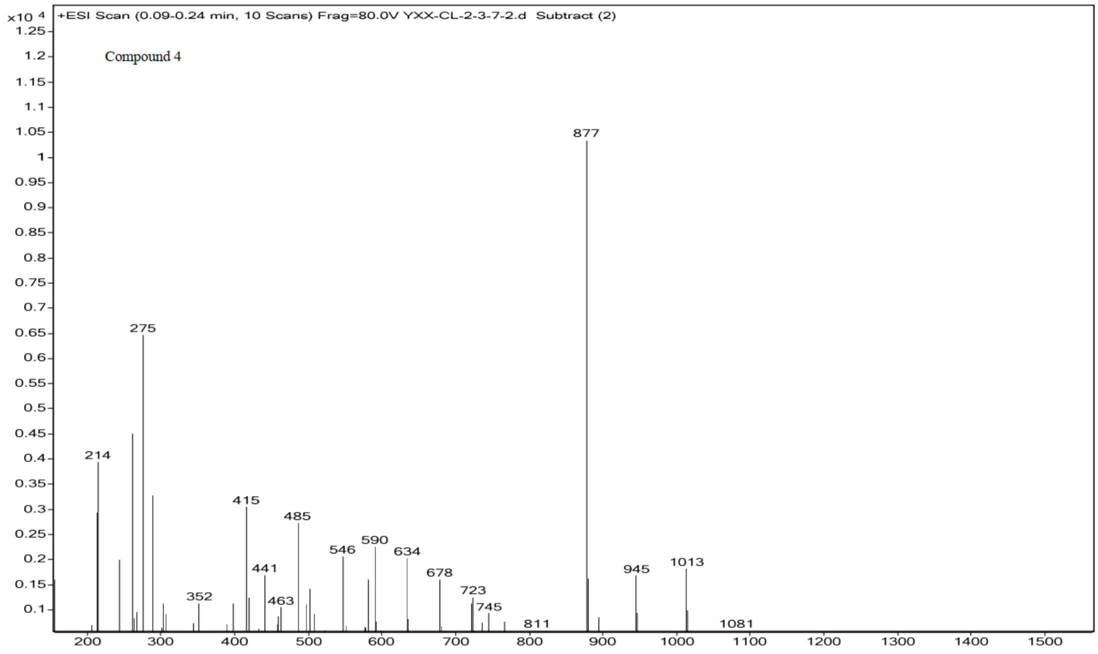

Figure S4-3 ESI-MS Spectra for Saponin **4**.

5. Saponin 5

Saponin **5**: White solid, $C_{51}H_{82}O_{21}$; CAS No., 68124-04-9; Paris saponin VII;
R_f = 0.2 ($CH_2Cl_2/CH_3OH/CH_3COOH$ = 8/2/0.1).
^{1}H-NMR (600 MHz, C_5D_5N) δ: 0.46 (3H, d, CH_3-27), 0.74 (3H, s, CH_3-18), 0.86 (3H, s, CH_3-19), 1.01 (3H, d, CH_3-21), 1.38 (3H, d, CH_3-Rha II), 1.38 (3H, d, CH_3-Rha III), 1.56 (3H, d, CH_3-Rha I), 4.64 (1H, d, Glc-H-1), 5.07 (1H, br, H-6), 5.63 (1H, s, Rha II-H-1), 6.08 (1H, s, Rha III-H-1), 6.20 (1H, s, Rha I-H-1), (See Figure S5-1).
^{13}C-NMR (600 MHz, C_5D_5N) δ: 141.5 (C-5), 122.6 (C-6), 110.5 (C-22), 104.1 (Rha II-C1), 102.9 (Rha I-C1), 102.9 (Rha III-C1), 101.0 (Glc-C1), 90.9 (C-16), 90.7 (C-17), 81.1 (Rha II-C4), 78.7 (C-3), 78.6 (Glc-C2), 78.5 (Glc-C4), 78.3 (Glc-C3), 77.7 (Glc-C5), 74.9 (Rha I-C4), 74.8 (Rha III-C4), 74.0 (Rha II-C3), 73.6 (Rha I-C3), 73.6 (Rha III-C3), 73.6 (Rha II-C2), 73.4 (Rha III-C2), 73.3 (Rha I-C2), 71.2 (Rha III-C5), 70.3 (Rha I-C5), 69.0 (Rha II-C5), 67.4 (C-26), 61.9 (Glc-C6), 53.8 (C-14), 51.0 (C-9), 45.9 (C-13), 45.5 (C-20), 39.7 (C-4), 38.3 (C-1), 38.3 (C-12), 37.9 (C-10), 33.2 (C-7), 33.1 (C-15), 32.8 (C-23), 32.5 (C-8), 31.2 (C-25), 30.9 (C-2), 29.5 (C-24), 21.7 (C-11), 20.2 (C-19), 19.6 (Rha III-C6), 19.4 (Rha I-C6), 19.2 (Rha II-C6), 18.0 (C-27), 17.9 (C-18), 10.5 (C-21), (See Figure S5-2).
ESI-MS *m/z* 1053 $[M+Na]^+$ (See Figure S5-3).

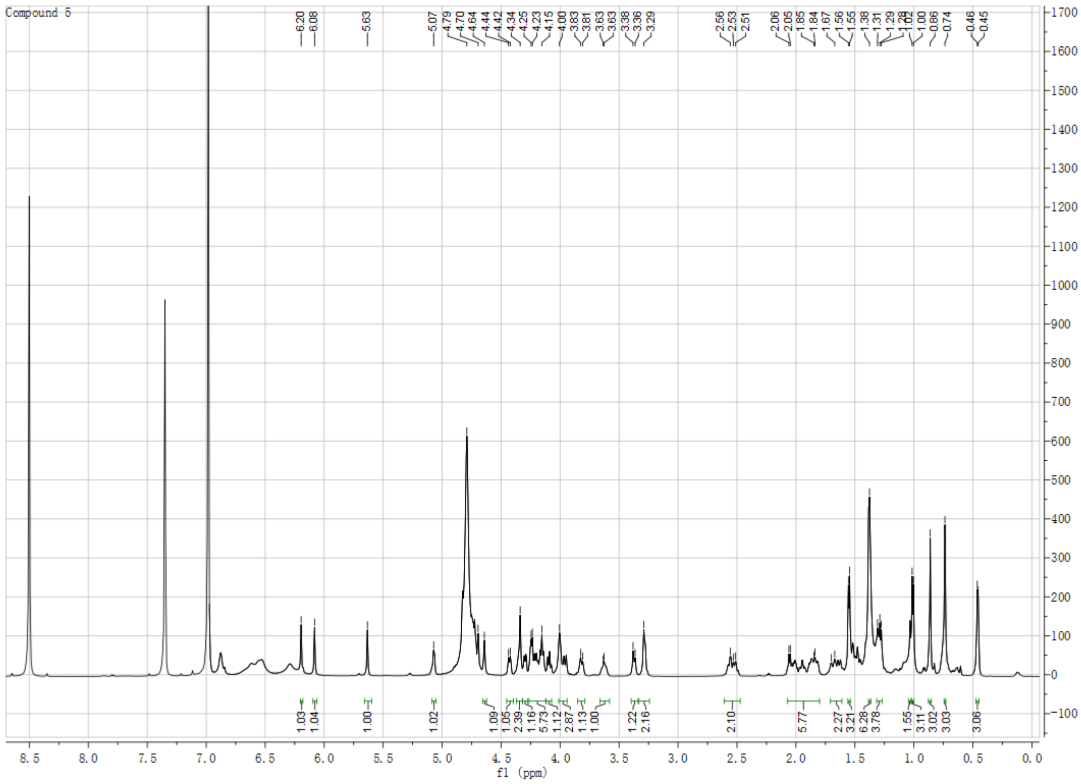

Figure S5-1 ^{1}H NMR Spectra for Saponin **5**.

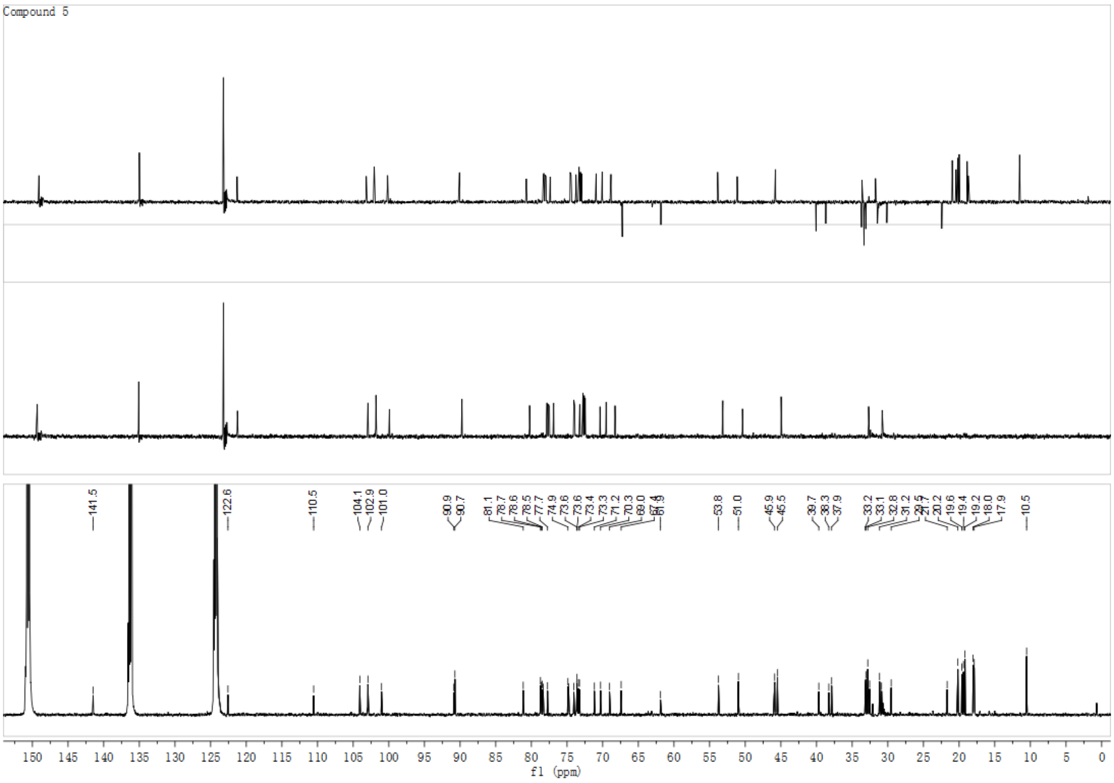

Figure S5-2 ^{13}C NMR Spectra for Saponin **5**.

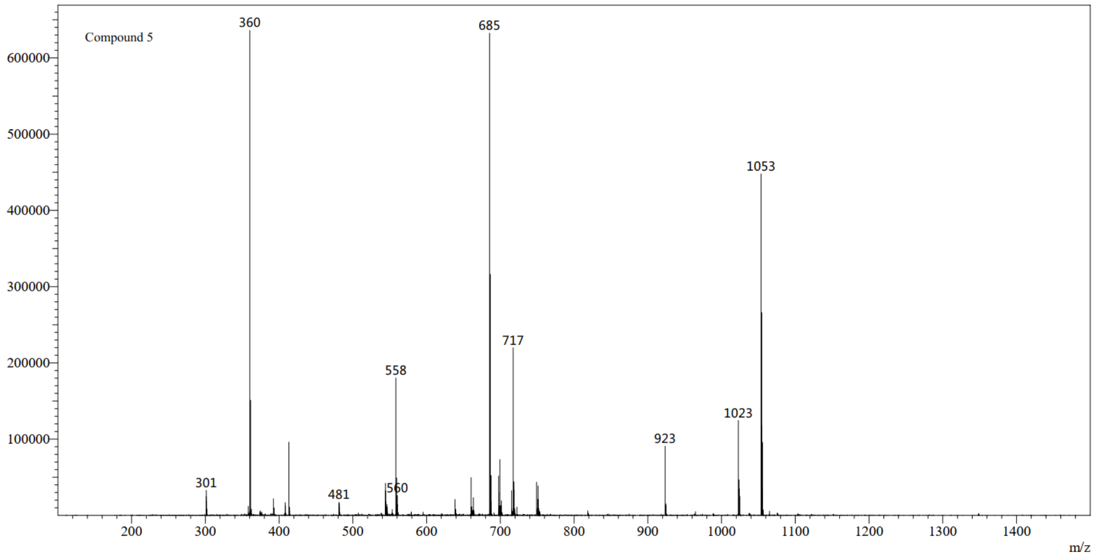

Figure S5-3 ESI-MS Spectra for Saponin **5**.

6. Saponin 6

Saponin **6**: White solid, $C_{51}H_{82}O_{20}$; CAS No., 50773-42-7; Paris saponin II;
R_f = 0.2 (CH_2Cl_2/CH_3OH/CH_3COOH = 8/2/0.1)
^{1}H-NMR (600 MHz, C_5D_5N) δ: 0.47 (3H, d, CH_3-27), 0.60 (3H, s, CH_3-18), 0.83 (3H, s, CH_3-19), 0.92 (3H, d, CH_3-21), 1.38 (3H, d, CH_3-Rha II), 1.39 (3H, d, CH_3-Rha III), 1.56 (3H, d, CH_3-Rha I), 4.44 (1H, d, Glc-H-1), 5.09 (1H, br, H-6), 5.64 (1H, s, Rha II-H-1), 6.08 (1H, s, Rha III-H-1), 6.20 (1H, s, Rha I-H-1), (See Figure S6-1).
^{13}C-NMR (600 MHz, C_5D_5N) δ: 141.5 (C-5), 122.5 (C-6), 110.0 (C-22), 104.1 (Rha III-C1), 102.9 (Rha I-C1), 102.9 (Rha II-C1), 101.0 (Glc-C1), 81.8 (C-16), 81.1 (Rha II-C4), 78.7 (C-3), 78.6 (Glc-C2), 78.5 (Glc-C4), 78.3 (Glc-C3), 77.7 (Glc-C5), 74.9 (Rha I-C4), 74.7 (Rha III-C4), 74.0 (Rha II-C3), 73.6 (Rha II-C2), 73.6 (Rha I-C3), 73.6 (Rha III-C3), 73.4 (Rha III-C2), 73.2 (Rha I-C2), 71.2 (Rha III-C5), 70.3 (Rha I-C5), 69.0 (Rha II-C5), 67.6 (C-26), 63.6 (C-17), 61.9 (Glc-C6), 57.3 (C-14), 51.0 (C-9), 42.7 (C-20), 41.2 (C-13), 40.6 (C-12), 39.7 (C-4), 38.2 (C-1), 37.9 (C-10), 32.9 (C-7), 32.5 (C-15), 32.4 (C-23), 31.3 (C-8), 30.9 (C-25), 30.7 (C-2), 30.0 (C-24), 21.8 (C-11), 20.1 (C-19), 19.6 (Rha II-C6), 19.4 (Rha I-C6), 19.2 (Rha III-C6), 18.0 (C-27), 17.1 (C-18), 15.8 (C-21), (See Figure S6-2).
ESI-MS *m/z* 1037 $[M+Na]^+$ (See Figure S6-3).

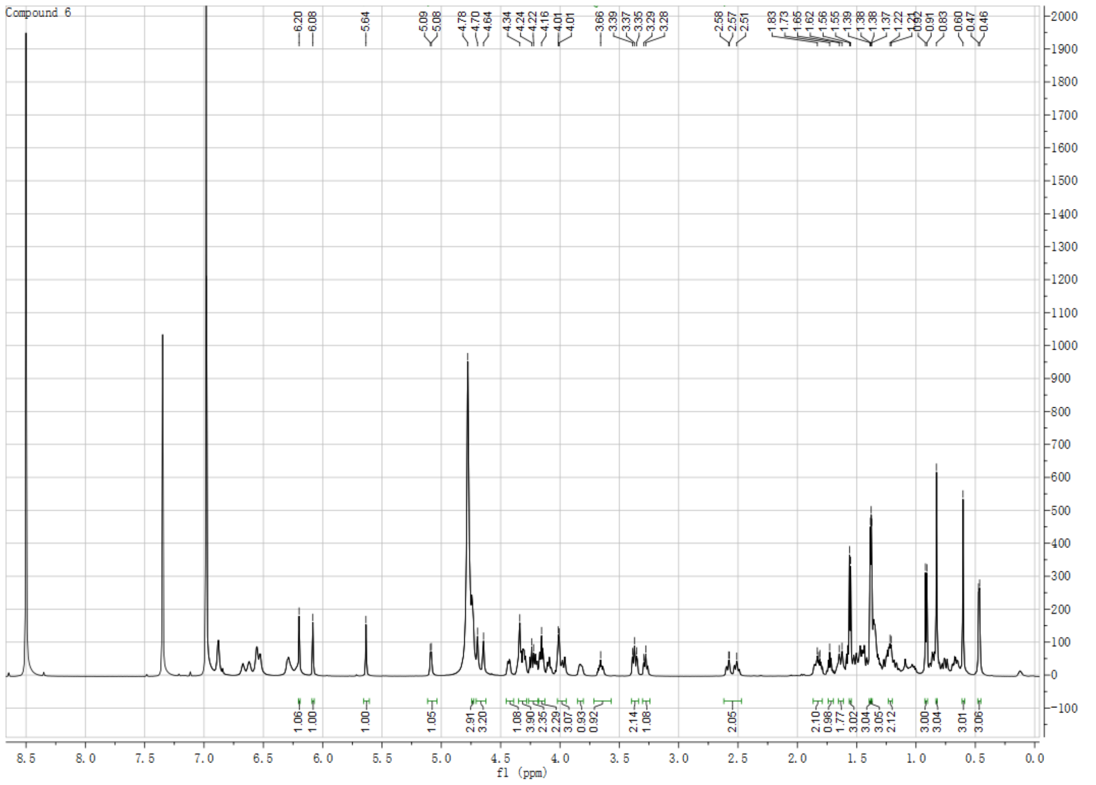

Figure S6-1 ^{1}H NMR Spectra for Saponin **6**.

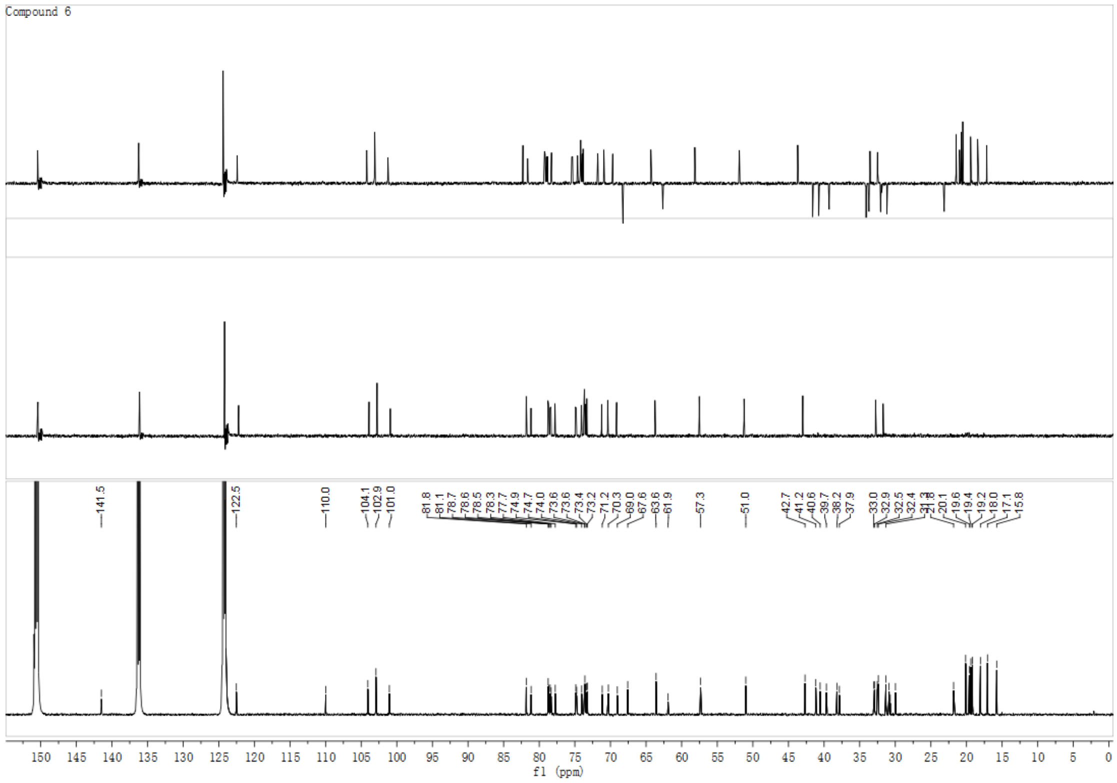

Figure S6-2 ^{13}C NMR Spectra for Saponin **6**.

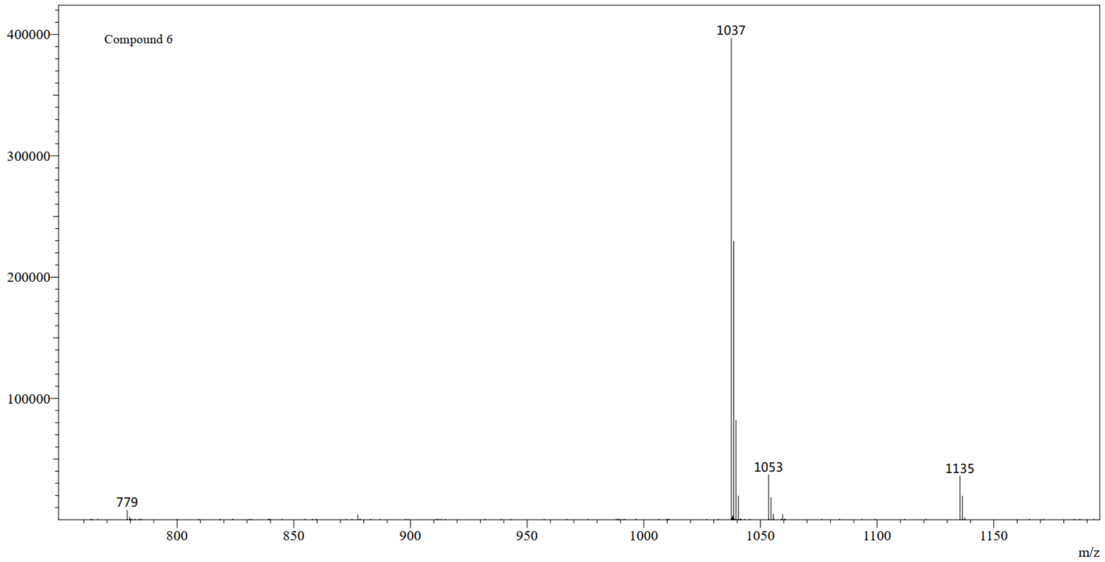

Figure S6-3 ESI-MS Spectra for Saponin **6**.
